# Developmental trajectories of internalizing problems among individuals born very preterm/very low birthweight: early risk and resilience factors

**DOI:** 10.1007/s00787-025-02736-3

**Published:** 2025-05-15

**Authors:** Yanlin Zhou, Peter Bartmann, Nicole Tsalacopoulos, Dieter Wolke

**Affiliations:** 1 Department of Psychology, University of Warwick, University Road, Coventry, UK; 2 Division of Health Sciences, Warwick Medical School, University of Warwick, Coventry, UK; 3 Department of Neonatology and Paediatric Intensive Care, University Hospital Bonn, Children’s Hospital, Bonn, Germany; 4 School of Psychological Sciences, Monash University, Melbourne, Australia; 5 Department of Pediatrics, University Hospital Würzburg, Würzburg, Germany; 6 Department of Population Health Sciences, University of Leicester, Leicester, UK

**Keywords:** Very preterm, Very low birthweight, Internalizing behavior, Developmental trajectories, Risk and resilience

## Abstract

Individuals born very preterm (VPT; < 32 weeks) or with very low birthweight (VLBW; < 1500 g) are at higher risk for internalizing problems compared to those born at term (37–42 weeks) or with normal birthweight (> 2500 g). However, group-level comparisons often overlook individual differences within these populations. Using data from the Bavarian Longitudinal Study, a German population-based birth cohort, this study aims to investigate developmental trajectories of internalizing problems from childhood to adulthood in 368 VPT/VLBW individuals and to identify early-life neonatal, family, neurodevelopmental, and social factors associated with these trajectories. Growth mixture modeling of parent-reported internalizing problems at ages 6, 8, 13, and 26 revealed three distinct trajectories: 61.7% exhibited consistently low levels of internalizing problems, 21.7% showed increasing problems, and 16.6% showed decreasing problems over time. Compared to the consistently low group, the increasing group had lower socioeconomic status at birth, lower gestational age, and more neurosensory impairments; while the decreasing group was characterized by higher family adversity, greater shyness and emotionality, higher birthweight, and fewer parent–infant relationship problems. Multiple births were associated with a higher likelihood of exhibiting consistently low internalizing problems rather than the increasing or decreasing trajectories. Early interventions targeting higher-risk groups—such as those with lower gestational age, neurosensory impairments, socioeconomic disadvantages, family adversity, or challenging temperaments—and promoting resilience factors like positive parenting, have the potential to improve long-term mental health outcomes for VPT/VLBW individuals.

**Supplementary Information:**

The online version contains supplementary material available at 10.1007/s00787-025-02736-3.

## Introduction

Preterm birth (< 37 weeks gestation) and low birthweight (< 2500 g) account for approximately 10.6% and 14.6% of live births worldwide, respectively [1]. Very preterm (VPT; < 32 weeks) and very low birthweight (VLBW; < 1500 g) births represent 1–2% of newborns, with increased risks of medical complications and developmental challenges, including internalizing problems such as anxiety, depression, social withdrawal, and somatic complaints [2–4]. Advancements in neonatal care have significantly improved survival rates for VPT/VLBW infants, however, mental health outcomes for recent cohorts have not improved compared to those born before the millennium [5]. A meta-analysis of 10 international cohorts (1977–2004) showed VPT/VLBW individuals had twice the odds of anxiety disorders and 1.5 times the odds of mood disorders compared to their full-term or normal birthweight peers [6]. However, most studies focus on group-level mean comparisons, overlooking the heterogeneity within the VPT/VLBW group itself [7–11]. Investigating variability within the VPT/VLBW population by examining developmental trajectories of internalizing problems, and identifying related early biological and psychosocial profiles, can facilitate targeted interventions for high-risk subgroups [2, 12].

### Developmental continuity and heterogeneity

Internalizing problems can show homotypic continuity, where early symptoms predict similar issues later in life, or developmental heterogeneity, where early symptoms follow distinct patterns over time [13]. In VPT/VLBW populations, homotypic continuity is often observed, for example, VPT individuals experienced persistently higher levels of internalizing problems from ages 6 to 19 years compared to their full-term peers [8]. Similarly, VLBW adolescents continued to experience higher levels of internalizing problems into their thirties, while their normal-birthweight peers showed an age-related decline [14]. However, these studies typically used a “one size fits all” group-level mean comparisons, which fail to account for the heterogeneity within the VPT/VLBW group.

Studies using growth mixture modeling (GMM)—a method that considers both within-person changes and between-person variabilities—have identified three to five heterogenous developmental trajectories of internalizing problems during childhood and adolescence, with most individuals following a stable, low-symptom trajectory [15–21]. Specifically for preterm populations, three distinct trajectories were identified from ages 16 months to 6 years, showing consistently high (41%), moderate (42%), and low (17%) levels of internalizing problems, respectively [22]. However, how these developmental trajectories of internalizing problems unfold from childhood into adulthood in VPT/VLBW populations remains unknown, which is the first aim of this study.

### Early risk and resilience factors

Empirical findings and systematic reviews have identified many risk factors for higher internalizing problems in VPT/VLBW individuals, including biological factors (e.g., lower gestational age, underweight or small for gestational age), social factors (e.g., single-parent families, low caregiver education, parental stress), and individual factors (e.g., lower IQ, neurosensory impairments, regulatory problems) [22–25]. Despite these vulnerabilities, our understanding of developmental plasticity and resilience in VPT/VLBW individuals remains limited [26]. Only a few studies have examined resilience factors that protect VPT/VLBW individuals from internalizing problems, such as breastfeeding [27], positive parenting and delayed gratification—the ability to resist immediate temptations in favor of long-term rewards [28], maternal sensitivity [29], and peer support [25].

However, there remains a lack of studies systematically examining early-life factors that differentiate between risk (i.e., persistently high or increasing internalizing problems) and resilience (e.g., recovery or decreasing internalizing problems) outcomes in the long-term development of VPT/VLBW individuals. Therefore, this study adopts a multisystem perspective [30] to examine how neonatal and biological factors (e.g., gestational age, birthweight, medical complications), family and parenting (e.g., family relationships, parenting quality, family adversity), child neurodevelopment and temperament (e.g., cognitive skills, self-regulation), and social contexts (e.g., peer relationships, neighborhood quality) shape risk and resilience trajectories of internalizing problems in the VPT/VLBW population.

### Current study

This study has two aims: (1) to examine the heterogeneity in the developmental trajectories of internalizing problems within the VPT/VLBW population from childhood to adulthood, and (2) to identify the early risk and resilience factors associated with these trajectories, including neonatal and biological factors, family and parenting, child neurodevelopment and temperament, and social contexts.

## Methods

### Participants and design

The Bavarian Longitudinal Study (BLS, Bayerische Entwicklungs Studie) is a geographically defined, population-based birth cohort from Southern Bavaria, Germany, of infants born between January 1985 and March 1986. The cohort included all infants admitted to one of the 17 children’s hospitals within the first 10 days after birth (*N* = 7505; 10.6% of all live births) and 916 full-term controls in obstetric hospitals in the same region (i.e., 37–42 weeks of gestation, and not transferred to a pediatric hospital in the first 10 days after birth) [31]. Detailed descriptions of the BLS samples have been published previously [32], and are available through the RECAP database (https://platform.recap-preterm.eu/pub/study/best_bls).

**Fig. 1:**
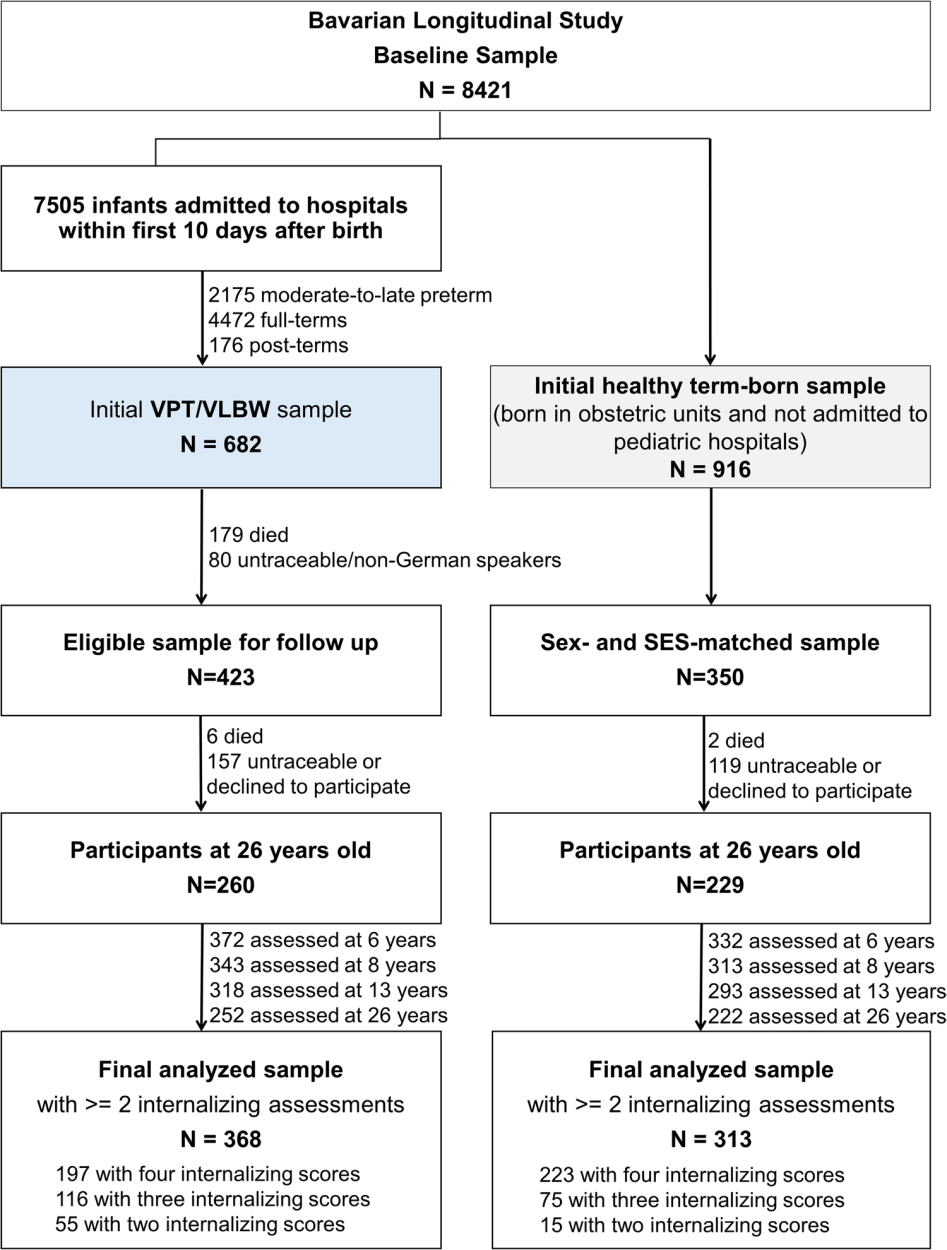
Flowchart of the very preterm and/or very low birth weight (VPT/VLBW) sample from the Bavarian Longitudinal Study. *Note.* Term-born controls were also followed up at the same time but were only included in the current analysis for z-standardizing scores across the whole sample

Figure 1 presents the flowchart of the current sample. Of the initial 682 VPT/VLBW infants, 508 survived and 411 were eligible (alive, German-speaking, residing in Germany) for the 26-year follow-up, with 260 (63.3%) participating. The final sample consisted of 368 VPT/VLBW participants with internalizing assessments available at least twice (197 with complete data, 116 with three, and 55 with two assessments). There were no significant differences in internalizing scores between participants with complete versus incomplete data across the four waves (Table S1). The final VPT/VLBW sample did not significantly differ from the 140 participants lost to follow-up after NICU discharge in sex, gestational age, birthweight, hospitalization duration, maternal age, parental marital status, or maternal mental health; however, those who dropped out were more likely to be from lower socioeconomic backgrounds (Table S2). Of the initial 916 healthy term-born children, 350 were randomly selected to match on sex and SES with the VPT/VLBW group. In adulthood, 229 participated at 26 years (see Fig. 1), with internalizing scores measured using the same assessment tools. The final sample of term-born controls, consisting of 313 participants (49.8% male; gestational age: M ± SD = 39.65 ± 1.17 weeks; birth weight: M ± SD = 3386 ± 450 g), included only those with internalizing data from at least two assessments waves. This group was used exclusively to generate standardized z-scores for internalizing problems across the entire sample.

### Procedure

Data were collected for both VPT/VLBW and full-term groups across four phases: Phase I (birth, 5 months, 20 months, 56 months), Phase II (ages 6 and 8 years), Phase III (age 13 years), and Phase IV (age 26 years). At birth, neonatal data and medical complications were documented daily during hospitalization by trained clinicians using standardized medical records. Structured parental interviews on child and family functioning were conducted shortly after birth and repeated at each subsequent time point. Follow-up developmental and neurological assessments at 5, 20, and 56 months were conducted in hospital-based laboratory settings by trained pediatricians and developmental neurologists, using standardized protocols. Cognitive development was assessed at 56 months using a battery of three standardized tasks. Internalizing problems were measured via parent-report questionnaires completed on the assessment day at ages 6 and 8, and through both self-report and parent-report questionnaires at ages 13 and 26. At age 13, questionnaires were distributed and returned by post. At age 26, self-report assessments were completed in person during laboratory visits, while parent-report questionnaires were sent by post and followed up with telephone reminders.

Parents of newborns were approached within 48 h of the infant’s hospital admission and provided written informed consent for participation. For adult follow-up assessments, all participants (or their legal representatives) provided fully informed written consent. The BLS study received ethical approval from the Ethics Committee of the University of Munich Children’s Hospital and the Bavarian Health Council (Landesärztekammer Bayern); approval for adult follow-up was granted by the Ethical Board of the University Hospital Bonn (Reference #159/09).

## Measures

### Internalizing problems from childhood to adulthood

At ages 6 and 8, internalizing problems were measured using the parent-report version of the German version Child Behavior Checklist (CBCL) [33]. Internalizing raw scores were calculated by summing the anxious/depressed, social withdrawal, and somatic complaints subscales. At age 13, both self-reports and parent reports were collected using the German version Strengths and Difficulties Questionnaire (SDQ) [34]. Internalizing raw scores were derived by summing the emotional problems and peer relationship problems subscales [35]. At age 26, participants completed the German version of the Young Adult Self-Report (YASR), while parents completed the German version of the Young Adult Behavior Checklist (YABCL). Internalizing raw scores were computed by summing the anxious/depressed and withdrawn subscales, following the original manual [36]. The SDQ and CBCL are comparable in distinguishing between community and clinic samples for internalizing domains [37, 38]. To ensure the construct validity across measures, Confirmatory Factor Analysis (CFA) was conducted for each parent report assessment, showing acceptable model fit (Figure S1). The four measures showed good internal consistency, with Cronbach’s α values of 0.81, 0.79, 0.81, and 0.92.

### Early risk and resilience factors

#### Neonatal and biological factors

Biological sex (male/female), gestational age (in completed weeks), birthweight (in grams), and multiple birth (singleton or multiple) were extracted from medical records. Birthweight was standardized using the Fenton growth chart, adjusted for sex and gestational age [39]. Medical complications, including intraventricular hemorrhage (i.e., severe bleeding into the ventricles grade 3 or 4) and >bronchopulmonary dysplasia, were based on clinical assessments neonatally and coded as binary variables. Breastfeeding (ever breastfed) was recorded as a binary variable from parental interviews at 5 months.

#### Family and parenting factors

*Socioeconomic status (SES)* at birth was determined using a weighted composite of parental occupation and education levels from interviews, categorized into three levels (1 = low, 2 = middle, 3 = high) [31, 40]. *Maternal mental health* during the neonatal period was assessed via the Maternal Psychiatric Interview [41] and coded as binary (0 = has mental health problems, 1 = no mental health problems). *Parent–infant relationship problems* were assessed neonatally through nurse observations and a standardized parental interview at 5 months using the Parent Infant Relationship Index (PIRI) [42],with eight dichotomous items covering attachment concerns and relationship problems ranging from 0 (no problems) to 8 (severe problems). Family adversity and psychosocial stress were repeatedly assessed through parental interviews [43] at 5, 20, and 56 months. *Family adversity *included eight items (e.g., early parenthood, single-parent status, poor household conditions), and a cumulative adversity score (0–24) was calculated across the three time points. *Psychosocial stress* was measured based on parent interview using 14 items covering parental stressful events (e.g., bereavement, work pressure, financial difficulties, marital problems, health concerns, social conflicts), with higher cumulative scores indicating greater stress. *Parenting quality* was rated by interviewers at age 6 based on observed parent–child interactions during the structured Mannheimer Parent Interview [44]. A single item assessed overall parenting quality (1 = very good to 5 = very bad). *Partnership quality *at age 6 was assessed using a modified Dyadic Adjustment Scale [45], a 38-item measure of satisfaction, cohesion, consensus, and affectional expression, with higher scores indicating better partnership quality.

#### Child neurodevelopment and temperament

*Cognitive ability* (IQ) at 56 months was assessed using a composite score from three cognitive tasks [46]: the Columbia Mental Maturity Scale [47], the Active Vocabulary Test [48], and the Beery-Buktenica Developmental Test of Visual-Motor Integration [49]. *Neurosensory impairment* (NSI) was coded as a binary variable, indicating any impairment of non-ambulatory cerebral palsy, blindness, uncorrected hearing loss, or low cognitive ability (IQ < −2 SD) at both 56 months and 6 years. *Regulatory problems* at age 6 were assessed during the Mannheimer Parent Interview, covering difficulties with falling asleep, maintaining sleep, and eating disorders, scored from 0 (no problems) to 3 (severe problems) [43]. *Child temperament* at age 6 was measured using the parent-reported Emotionality Activity and Sociability questionnaire [50], consisting of 37 items across five dimensions: activity, effortful control, shyness, sociability, and emotionality. Items were rated on a 5-point scale (1 = never to 5 = always), with higher scores indicating higher levels of each dimension.

#### Social contexts at age 6

*Peer relationships* were assessed using the 6-item subscale of the Perceived Competence Scale for Children [51], with higher scores indicating better peer interactions. *Friendship quality* was derived from three items—number of friends, best friends, and peer contact frequency—from a semi-structured Friendship and Family Interview [52, 53]. *Bully victimization* was assessed during the Mannheimer Parent Interview [44], using two questions about being “irritated, insulted, or bullied by other children” and “physically harmed by peers”, rated from 1 (never) to 7 (every day), with a higher total score indicate more frequent victimization. *Neighborhood child-friendliness* was rated by parents on a scale from 1 (not child-friendly) to 4 (very child-friendly).

### Data analyses

Discrepancies between parent and self-reports are well-documented [54, 55], and parents of VPT/VLBW children typically report more internalizing problems than the participants themselves [9, 56, 57]. In our VPT/VLBW sample, parent and self-reports at ages 13 and 26 showed moderate to strong correlations (*r*s = 0.49–0.60), supporting the validity of parent ratings. However, parents consistently reported higher levels of internalizing symptoms, with differences ranging from 0.23 to 0.30 standard deviations at each time point. This suggests that incorporating self-reports at later time points could introduce bias and compromise compatibility over time. Therefore, to maintain consistency and avoid informant-related bias, we used only parent reports in the current internalizing trajectory analysis. This analysis plan was pre-registered on the Open Science Framework (osf.io/wun9d).

First, parent-reported internalizing scores at ages 6, 8, 13, and 26 were standardized on the whole sample, including VPT/VLBW and their term controls. GMM [58] was conducted to examine developmental trajectories using the standardized scores. Missing data were handled via Full Information Maximum Likelihood in Mplus version 8. The mean and variance of the intercept (i.e., the estimated level of internalizing problems at age 6) were freely estimated. To ensure that individuals within the same class exhibited similar slopes and to enhance model convergence, the variance of the slope was fixed to zero within each class, while the slope means (i.e., the yearly change in internalizing problems) were freely estimated across classes. Models with 1 to 5 classes were tested, and better model fit and class separation were determined by lower Akaike Information Criterion (AIC) and Sample-Size Adjusted BIC (SSA-BIC), higher entropy (0 to 1), and a significant Vuong-Lo-Mendell-Rubin likelihood ratio test (VLMRT, *p* < 0.05 indicating preference for *k* over *k*−1 classes) [58]. Individuals were grouped into latent trajectory classes based on their highest posterior probability, and class membership was subsequently treated as a multinomial outcome in logistic regression models. Second, univariable logistic regression analyses, followed by multivariable regression accounting for covariates, was used to identify early factors differentiating risk and resilience trajectories. Odds ratios with 95% confidence intervals and *p*-values were reported. Missing values were imputed in the multivariable analysis using Multiple Imputation by Chained Equations (*mice* package) in R [59].

Post-hoc sensitivity analysis. Given the classification uncertainties in assigning individuals to their most likely class and the presence of 64 siblings clustered within 27 families, we conducted a post-hoc sensitivity analysis. In the multivariable multinomial regression, each individual’s posterior probability of class membership was used as a weight, while cluster-robust standard errors were applied to account for family clustering. The results are presented in Table S5 of the supplementary material.

## Results

### Final sample characteristics

Neonatal and sociodemographic characteristics of the 368 VPT/VLBW participants are in Table 1.

**Table 1:** Demographic characteristics of the VPT/VLBW sample (*n* = 368)

Male	192 (52.2)
Gestational age (week) [M (SD)]	30.49 (2.29)
Birthweight (gram) [M (SD)]	1294.72 (307.14)
Birthweight z score^a^ [M (SD)]	−0.73 (1.23)
Groups by birthweight z score	
* Small for gestation age (*< *10 th percentile)*	113 (30.7)
* Average for gestation age (10 th-90 th percentiles)*	243 (66.0)
* Large for gestation age (*> *90 th percentile)*	12 (3.3)
Multiple births	89 (24.2)
Medical complications	
* Severe intraventricular hemorrhage (grade 3–4)*	25 (6.8)
* Bronchopulmonary dysplasia*^b^	201 (54.6)
* Neurosensory impairments*^c^	103 (28.0)
Ever breastfed	82 (22.3)
Maternal age at birth [M (SD)]	28.6 (5.02)
Family SES at birth	
* High SES*	77 (20.9)
* Middle SES*	159 (43.2)
* Low SES*	132 (35.9)

Statistics are reported as *n* (%) unless otherwise specified. ^a^Birthweight was standardized using the Fenton growth chart, adjusted for sex and gestational age [39]. ^b^Bronchopulmonary dysplasia was defined by chest x-ray evidence or mechanical ventilation > 28 days. ^c^Neurosensory impairments included non-ambulatory cerebral palsy, blindness in one or both eyes, uncorrected hearing loss, or IQ below 2 standard deviations

### Determining trajectories of internalizing problems

**Fig. 2:**
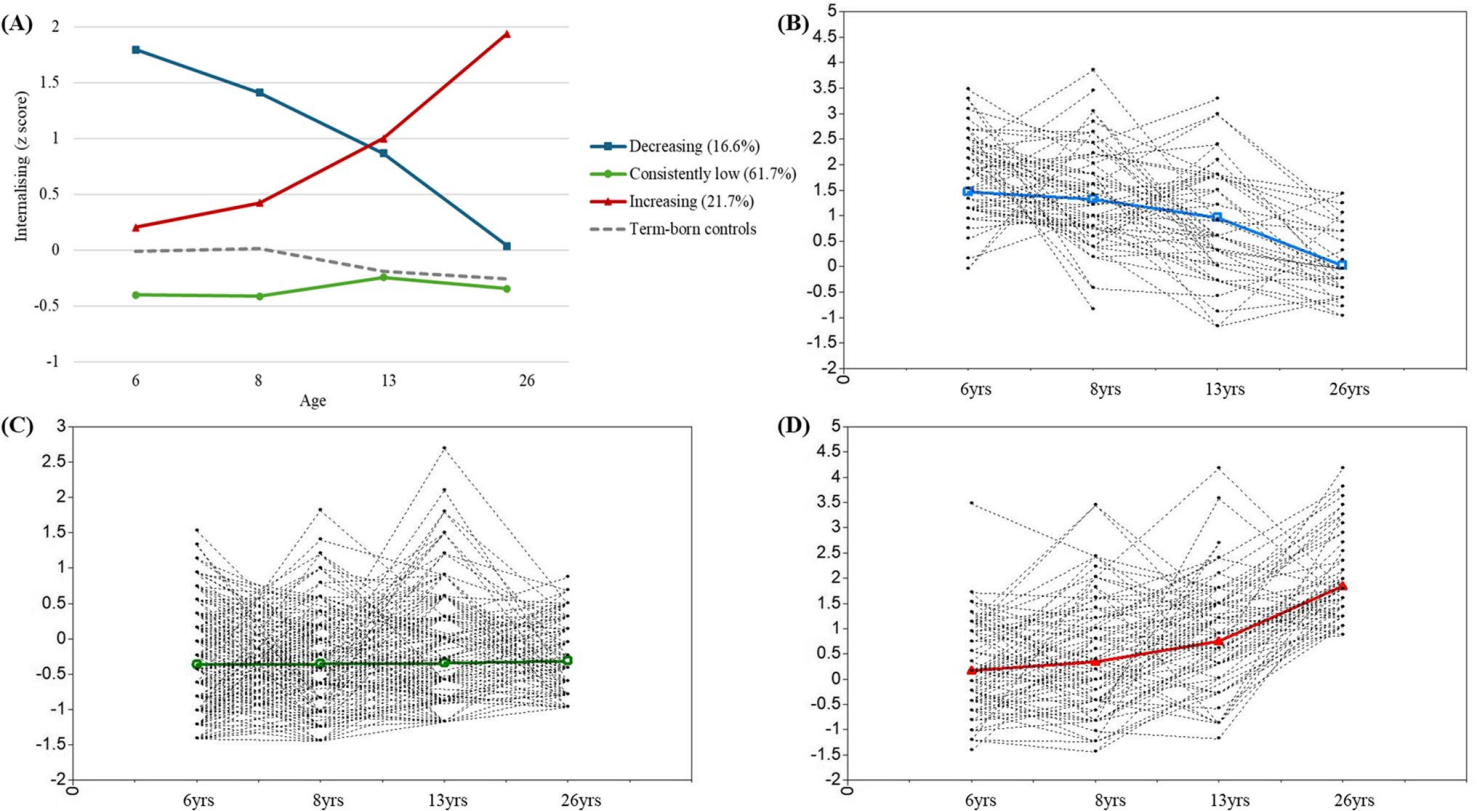
Trajectories of internalizing problems from ages 6 to 26. Panel **A** shows the observed mean levels of the three trajectories. Panels **B**, **C**, and **D** display estimated means and observed individual values for the Decreasing, Consistently Low, and Increasing trajectories, respectively

Table 2 presents model fit indices for the GMMs with 1 to 5 classes. The three-class solution was identified as the most parsimonious, supported by lower AIC and SSA-BIC, and a significant VLMR test. The three identified internalizing trajectories are labeled as consistently low, increasing, and decreasing. Table 3 presents the characteristics of the three trajectory groups, the consistently low group showed stably low internalizing problems with significant individual variability in initial levels but no significant change over time. The increasing group exhibited a significant rise in internalizing problems, with substantial variability in initial levels. The decreasing group began with high internalizing problems that declined significantly over time, with notable within-group variability in starting levels. Figure 2 illustrates group-level and individual growth for each trajectory group. Table S3 shows the proportion of clinical categories for internalizing scores at each timepoint across trajectories. The increasing group had a rising percentage of individuals classified as borderline or in clinical categories over time, whereas the decreasing group exhibited a decline in these classifications.

**Table 2:** Model fit indices for the GMM of internalizing problems from 1 to 5 classes

Class	AIC	SSA-BIC	VLMR (*p*)	Entropy	*n*%_[c1]_	*n*%_[c2]_	*n*%_[c3]_	*n*%_[c4]_	*n*%_[c5]_
1	3574	3579	-	-	-				
2	3528	3536	0.026	0.67	22.6%	77.4%			
**3**	**3462**	**3473**	** < 0.001**	**0.66**	**16.6%**	**21.7%**	**61.7%**		
4	3448	3462	0.406	0.63	6.3%	16.6%	22.3%	54.9%	
5	3442	3459	0.198	0.68	6%	7.1%	12.2%	17.9%	56.8%

*AIC* Akaike’s Information Criterion; *SSA-BIC* Sample-size Adjusted Bayesian Information Criterion; *VLMRT* Vuong-Lo-Mendell-Rubin Likelihood Ratio Test

Bolded values indicate the best-fitted model

**Table 3:** Characteristics of growth mixture model trajectories

		Intercept				Slope	
Trajectories	*N* (%)	Mean	*p*	Variance	*p*	Mean	*p*
Consistently low	227 (61.7%)	−0.366	< 0.001	0.064	0.008	0.003	0.566
Increasing	80 (21.7%)	0.170	0.157	0.445	< 0.001	0.084	< 0.001
Decreasing	61 (16.6%)	1.465	< 0.001	0.266	< 0.001	−0.072	< 0.001

The variance of the slope was fixed at 0 in each group, constraining individual slopes within each group to be similar. The slope unit represents change per year

### Early factors differentiate these internalizing trajectories

**Fig. 3:**
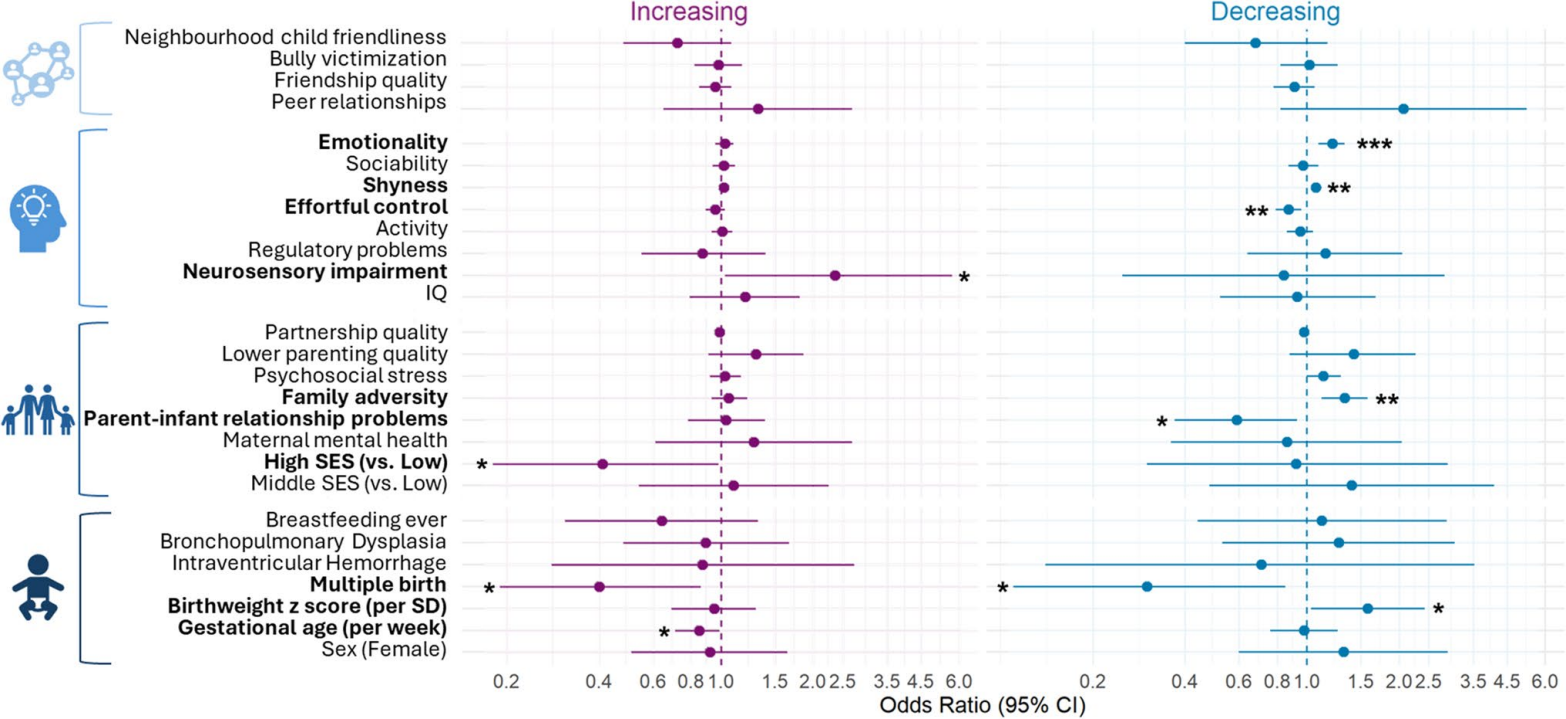
Multivariable associations of early factors with increasing and decreasing internalizing trajectories (consistently low trajectory as reference). Odds ratio and 95% confidence intervals (CI) are shown. ^*^p < 0.05, ^**^p < 0.01, ^***^p < 0.001. Bolded factors indicate significant associations with the Increasing or Decreasing trajectories. The association between effortful control and membership in the decreasing trajectory was no longer observed in the sensitivity analysis that accounted for classification uncertainty and family cluster-robust standard errors

Figure S2 presents bivariate correlations among early factors, and Figure S3 details their missing patterns. Table S4 shows the univariable and multivariable multinomial logistic regression results for associations between early factors and memberships in increasing or decreasing trajectories, with consistently low trajectory as the reference. Multivariable results (Fig. 3) showed that the increasing trajectory was characterized by lower gestational age (OR = 0.83 [0.7, 0.99], *p* = 0.046), lower likelihoods of multiple births (OR = 0.43 [0.20, 0.93], *p* = 0.032), lower SES at birth (OR = 0.41 [0.18, 0.98], *p* = 0.045), and higher likelihood of NSI (OR = 2.47 [1.03, 5.92], *p* = 0.044). Decreasing internalizing problems were associated with higher standardized birthweight (OR = 1.61 [1.05, 2.46], *p* = 0.029), lower likelihoods of multiple births (OR = 0.31 [0.11, 0.87], *p* = 0.027), fewer parent–infant relationship problems (OR = 0.60 [0.38, 0.94], *p* = 0.030), greater family adversity (OR = 1.32 [1.11, 1.57], *p* < 0.001), lower effortful control (OR = 0.87 [0.79, 0.96], p = 0.005), higher shyness (OR = 1.07 [1.03, 1.12], *p* = 0.002), and higher emotionality (OR = 1.20 [1.09, 1.32], *p* < 0.001). However, the association between effortful control and membership in the decreasing trajectory was no longer observed in the sensitivity analysis that accounted for classification uncertainty and family cluster-robust standard errors.

## Discussion

We identified three distinct developmental trajectories of internalizing problems—increasing, decreasing, and consistently low—among individuals born VPT/VLBW from childhood to early adulthood. Our results demonstrate that VPT/VLBW children follow multiple distinct developmental paths, which are associated with a combination of neonatal, family, and neurodevelopmental factors. Compared to those with consistently low internalizing problems, individuals in the increasing trajectory were more likely to have lower SES at birth, lower gestational age, and early childhood NSI; while individuals in the decreasing trajectory experienced greater family adversity, had higher levels of shyness and emotionality, but had higher birthweight and fewer parent–infant relationship problems. Multiple births were associated with a higher likelihood of following a consistently low trajectory, rather than increasing or decreasing trajectories. These findings emphasize the heterogeneity of development within the VPT/VLBW population, with most individuals showing low internalizing problems over time.

The three trajectories of internalizing problems align with previous findings using the same GMM analytical method in general child samples [17, 20, 21]. Our study extends this research by examining development from childhood to adulthood, capturing variability within the high-risk VPT/VLBW population, and offering insights beyond group-level or static mean comparisons. Notably, we found a higher proportion in the increasing trajectory (21.7%), compared to the 6–13% reported in general population samples [17, 20, 21]. This may reflect the greater biological vulnerabilities and early-life disadvantages faced by VPT/VLBW individuals [60, 61], as our findings indicate that those with lower gestational ages and NSI were more likely to follow an increasing trajectory. Additionally, our extended follow-up over 20 years captures adolescence, a period often marked by intensifying internalizing problems due to increased autonomy, complex peer relationships, and rapid physical and social changes [62].

Our results provide insights into the early-life profiles associated with these developmental trajectories, highlighting key risk and resilience factors at individual and family levels. Lower family SES at birth, lower gestational age, and the presence of NSI were associated with increasing internalizing problems. Lower family SES is commonly linked to a heightened risk of emotional challenges in both general [63] and preterm populations [22]. This underscores the critical role of early economic hardship in development, especially since VPT/VLBW individuals are more often born into lower-income households due to the socioeconomic inequalities in preterm birth incidence [64]. In the VPT/VLBW population, lower gestational age remains a significant risk factor for increasing internalizing problems, illustrating a dose–response relationship between prematurity and developmental challenges, and the importance of early interventions for those at high risk [65, 66]. Despite advances in obstetric and neonatal care that have improved survival rates for VPT/VLBW infants, NSI remains prevalent, even in contemporary birth cohorts [67, 68]. In our sample, up to 28% of VPT/VLBW individuals had severe NSI, significantly impacting socioemotional functioning and often leading to low self-esteem, peer relationship difficulties, social isolation, and emotional disorders [24]. These findings emphasize the lasting impact of early socioeconomic disadvantages, compounded by lower gestational age and NSI, on emotional vulnerability and the elevated risk of increasing internalizing problems over time.

Decreasing internalizing problems were associated with higher family adversity, challenging temperaments in childhood (e.g., high shyness and emotionality), but also higher birthweight and fewer parent–infant relationship problems. The relationship between higher family adversity and decreasing internalizing problems is complex. One explanation is a potential “steeling effect,” where early family stress fosters resilience, helping individuals cope better over time [69]. Alternatively, schooling may have provided these individuals with a daily escape from family hardships, and along with supportive parenting, opportunities to regulate behavior and emotions, leading to improvements during adolescence and adulthood. While shyness, emotionality, and dysregulation in childhood often signal later internalizing problems, some children with these temperaments improve as they grow, adapt to new social environments, and develop self-regulation strategies [70]. Longitudinal studies from large community samples have shown that most very shy or inhibited children do not develop anxiety, and most adolescents with anxiety disorders were not particularly shy as young children [71]. Additionally, research indicates that children with difficult temperaments benefit substantially from warm and supportive parenting, which leads to better socioemotional outcomes [72, 73]. These findings underscore the importance of person-environment interactions in understanding the development of internalizing problems. Specifically, we identified protective effects of higher birthweight and better parent–child relationship quality on long-term mental health outcomes for VPT/VLBW individuals, even amidst early psychosocial adversity. This extends previous research focused on internalizing problems at single time points [74, 75] and highlights the value of family-focused interventions, particularly those aimed at strengthening early parenting to support positive outcomes in VPT/VLBW populations.

Consistently low internalizing problems were associated with favorable health conditions (e.g., higher gestational age, absence of NSI), supportive family environments (e.g., lower family adversity, higher SES), and easier temperaments (e.g., lower shyness, lower emotionality). This aligns with prior findings that stable low-level behavior problems are linked to higher gestational age, better child regulation, lower maternal depression, and fewer socioeconomic challenges [22]. We also found that VPT/VLBW individuals from multiple births were more likely to maintain consistently low levels of internalizing problems, rather than following increasing or decreasing trajectories. Similarly, previous research showed that being part of a multiple birth reduces the risk of internalizing problems in moderately-late preterm children at age 4 [76]. This protective effect may stem from having a co-twin, offering companionship, emotional support, and shared experiences [77]. Overall, these findings support a multisystemic perspective of resilience, suggesting that positive outcomes arise from interactions across multiple factors, such as biological characteristics, caregiving environments, and individual traits, rather than a single driving factor [30].

There have been significant advancements in neonatal care over the past forty years and survival of VPT/VLBW has increased significantly. In contrast, serious morbidity such as IVH has not significantly declined over time [78] and the quality of survival in terms of mental health has not improved [5, 79]. Furthermore, recent meta-analyses suggest that family-centered care and new neonatal treatment approaches show only short-term benefits, with no lasting effects on outcomes beyond the toddler years [80]. Given this evidence, our findings from individuals born in the 1980 s’ remain relevant to the contemporary VPT/VLBW population, as they are not primarily driven by neonatal treatment improvements. However, we acknowledge that secular trends—such as changes in mental health prevalence due to broader societal factors—may impact all children, regardless of birth status. Nevertheless, such secular trends may alter their prevalence but are unlikely to alter the longitudinal associations observed in our study [81].

### Limitations and future directions

First, although the BLS offers a rich dataset covering key developmental periods from birth to adulthood, the generalizability of findings from this German cohort warrants replication in other international samples. Second, although the final sample size (*n* = 368) is relatively large for VPT/VLBW cohort studies over a 26-year period, it remains small compared to general population samples. This may limit the model’s ability to distinguish latent classes, leading to classification uncertainty. Additionally, the small subgroup sizes for the increasing and decreasing trajectories reduce statistical power to detect early risk and resilience factors. Although the final sample included diverse SES backgrounds (Low: 36%, Mid: 43.1%, High: 21%), it had a higher overall SES than those who dropped out (58.6% from lower SES). This selective dropout is common in long-term longitudinal studies of VPT/VLBW, however simulations indicate it may have limited impact on longitudinal associations [81, 82]. Third, while relying solely on parent reports ensures informant consistency for trajectory analysis, psychological research has long emphasized the value of multi-informant approaches. Discrepancies between informants may reflect more than mere measurement error and can offer meaningful insights into different aspects of a child’s behavior and context [9, 54, 55]. Future research may incorporate multiple informants (e.g., self, parents, teachers, and peers) to capture the developmental trajectory of internalizing problems from more comprehensive and diverse perspectives over time. Lastly, while we identified key risk and resilience factors at neonatal, family, and neurodevelopmental levels for the developmental trajectories of internalizing problems, other important factors, such as genetic influences, were not examined.

## Conclusion

Individuals born VPT/VLBW are at increased risk for internalizing problems, yet they exhibited distinct developmental trajectories from childhood to early adulthood. Most maintain consistently low levels of internalizing problems, some show gradual decreases with age, while others experience a continuous increase over time. Key risk factors for increasing internalizing problems include lower gestational age, NSI, and low SES, suggesting these children may benefit from regular monitoring and early support to prevent mental health deterioration. Conversely, higher birthweight and positive parent–infant relationships serve as protective factors, contributing to decreases in internalizing problems even amid higher familial and psychosocial challenges. Therefore, providing parenting support could be a valuable intervention for stressed families, helping to alleviate initially high internalizing problems in their VPT/VLBW children.

## Supplementary Information

Below is the link to the electronic supplementary material.

Supplementary file1 (DOCX 1043 KB)

## Data Availability

The data is not publicly accessible and is not available to external researchers. However, access may be requested by contacting the corresponding author D.W. or apply via the RECAP Preterm Cohort Platform (https://platform.recap-preterm.eu/pub/study/best_bls).

## References

[1] WHO (2022) Recommendations for care of the preterm or low birth weight infant. World Health Organization, Geneva 36449655

[2] Wolke D, Johnson S, Mendonça M (2019) The life course consequences of very preterm birth. Annu Rev Dev Psychol 1:69–92. 10.1146/annurev-devpsych-121318-084804

[3] Saigal S, Morrison K, Schmidt LA (2020) Health, wealth and achievements of former very premature infants in adult life. Semin Fetal Neonatal Med 25. 10.1016/j.siny.2020.101107 10.1016/j.siny.2020.101107 32312673

[4] Ritchie K, Bora S, Woodward LJ (2015) Social development of children born very preterm: A systematic review. Dev Med Child Neurol 57:899–918. 10.1111/dmcn.12783 25914112 10.1111/dmcn.12783

[5] Larsen J, Kochhar P, Wolke D et al (2023) Comparing behavioural outcomes in children born extremely preterm between 2006 and 1995: the EPICure studies. Eur Child Adolesc Psychiatry. 10.1007/s00787-023-02258-w 37430147 10.1007/s00787-023-02258-w PMC11098736

[6] Anderson PJ, de Miranda DM, Albuquerque MR et al (2021) Psychiatric disorders in individuals born very preterm / very low-birth weight: An individual participant data (IPD) meta-analysis. EClinicalMedicine 42:101216. 10.1016/j.eclinm.2021.101216 34901794 10.1016/j.eclinm.2021.101216 PMC8639417

[7] Lærum AMW, Reitan SK, Evensen KAI et al (2019) Psychiatric symptoms and risk factors in adults born preterm with very low birthweight or born small for gestational age at term. BMC Psychiatry 19:223. 10.1186/s12888-019-2202-8 31315591 10.1186/s12888-019-2202-8 PMC6636134

[8] Linsell L, Johnson S, Wolke D et al (2019) Trajectories of behavior, attention, social and emotional problems from childhood to early adulthood following extremely preterm birth: a prospective cohort study. Eur Child Adolesc Psychiatry 28:531–542. 10.1007/s00787-018-1219-8 30191335 10.1007/s00787-018-1219-8 PMC6445809

[9] Mathewson KJ, Chow CHT, Dobson KG et al (2017) Mental health of extremely low birth weight survivors: A systematic review and meta-analysis. Psychol Bull 143:347–383. 10.1037/bul0000091 28191983 10.1037/bul0000091

[10] Pyhälä R, Wolford E, Kautiainen H et al. (2017) Self-reported mental health problems among adults born preterm: A meta-analysis. Pediatrics 139. 10.1542/peds.2016-2690 10.1542/peds.2016-2690 28283612

[11] Robinson R, Lahti-Pulkkinen M, Schnitzlein D et al (2020) Mental health outcomes of adults born very preterm or with very low birth weight: A systematic review. Semin Fetal Neonatal Med 25:101113. 10.1016/j.siny.2020.101113 32402835 10.1016/j.siny.2020.101113

[12] Wouldes TA (2022) Fostering resilience to very preterm birth through the caregiving environment. JAMA Netw Open 5:E2238095. 10.1001/jamanetworkopen.2022.38095 36269362 10.1001/jamanetworkopen.2022.38095

[13] Speranza AM, Liotti M, Fortunato A (2023) Heterotypic and homotypic continuity in psychopathology : a narrative review. Front Psychol 1–15. 10.3389/fpsyg.2023.1194249 10.3389/fpsyg.2023.1194249 PMC10307982 37397301

[14] Van Lieshout RJ, Ferro MA, Schmidt LA et al (2018) Trajectories of psychopathology in extremely low birth weight survivors from early adolescence to adulthood: a 20-year longitudinal study. J Child Psychol Psychiatry Allied Discip 59:1192–1200. 10.1111/jcpp.12909 10.1111/jcpp.12909 PMC6193866 29667718

[15] Bista S, Tait RJ, Straker LM et al (2024) Joint developmental trajectories of internalizing and externalizing problems from mid-childhood to late adolescence and childhood risk factors: Findings from a prospective pre-birth cohort. Dev Psychopathol 1–16. 10.1017/S0954579423001505 10.1017/S0954579423001505 38174409

[16] Flouri E, Papachristou E, Midouhas E et al (2018) Early adolescent outcomes of joint developmental trajectories of problem behavior and IQ in childhood. Eur Child Adolesc Psychiatry 27:1595–1605. 10.1007/s00787-018-1155-7 29663072 10.1007/s00787-018-1155-7 PMC6245124

[17] Katsantonis I, Symonds JE (2023) Population heterogeneity in developmental trajectories of internalising and externalising mental health symptoms in childhood: differential effects of parenting styles. Epidemiol Psychiatr Sci 32. 10.1017/S2045796023000094 10.1017/S2045796023000094 PMC10130732 36999252

[18] Nivard MG, Lubke GH, Dolan CV et al (2017) Joint developmental trajectories of internalizing and externalizing disorders between childhood and adolescence. Dev Psychopathol 29:919–928. 10.1017/S0954579416000572 27427290 10.1017/S0954579416000572

[19] Papachristou E, Flouri E (2020) Distinct developmental trajectories of internalising and externalising symptoms in childhood: Links with mental health and risky behaviours in early adolescence. J Affect Disord 276:1052–1060. 10.1016/j.jad.2020.07.130 32768877 10.1016/j.jad.2020.07.130

[20] Parkes A, Sweeting H, Wight D (2016) Early childhood precursors and school age correlates of different internalising problem trajectories among young children. J Abnorm Child Psychol 44:1333–1346. 10.1007/s10802-015-0116-6 26747450 10.1007/s10802-015-0116-6 PMC5007267

[21] Sterba SK, Printein MJ, Cox MJ (2007) Trajectories of internalizing problems across childhood: Heterogeneity, external validity, and gender differences. Dev Psychopathol 19:345–366. 10.1017/S0954579407070174 17459174 10.1017/S0954579407070174

[22] Gerstein ED, Woodman AC, Burnson C et al (2017) Trajectories of externalizing and internalizing behaviors in preterm children admitted to a neonatal intensive care unit. J Pediatr 187:111–118. 10.1016/j.jpeds.2017.04.047 28533035 10.1016/j.jpeds.2017.04.047 PMC5533642

[23] Linsell L, Malouf R, Johnson S et al (2016) prognostic factors for behavioral problems and psychiatric disorders in children born very preterm or very low birth weight: A systematic review. J Dev Behav Pediatr 37:88–102. 10.1097/DBP.0000000000000238 26703327 10.1097/DBP.0000000000000238 PMC5330463

[24] Montagna A, Nosarti C (2016) Socio-emotional development following very preterm birth: Pathways to psychopathology. Front Psychol 7. 10.3389/fpsyg.2016.00080 10.3389/fpsyg.2016.00080 PMC4751757 26903895

[25] Jaekel J, Heinonen K, Baumann N et al (2023) Associations of crying, sleeping, and feeding problems in early childhood and perceived social support with emotional disorders in adulthood. BMC Psychiatry 23:1–9. 10.1186/s12888-023-04854-1 37268881 10.1186/s12888-023-04854-1 PMC10239120

[26] Wolke D (2018) Preterm birth: high vulnerability and no resiliency? Reflections on van Lieshout et al. (2018). J Child Psychol Psychiatry Allied Discip 59:1201–1204. 10.1111/jcpp.12971 10.1111/jcpp.12971 30339283

[27] Rodrigues C, Zeitlin J, Carvalho AR et al (2022) Behavioral and emotional outcomes at preschool age in children born very preterm: The role of breast milk feeding practices. Early Hum Dev 165. 10.1016/j.earlhumdev.2021.105535 10.1016/j.earlhumdev.2021.105535 35038626

[28] Poehlmann-Tynan J, Gerstein ED, Burnson C et al (2014) Risk and resilience in preterm children at age 6. Dev Psychopathol 760:843–858. 10.1017/S095457941400087X 10.1017/S095457941400087X PMC4834243 25196017

[29] Faure N, Habersaat S, Harari MM et al (2017) Maternal sensitivity: a resilience factor against internalizing symptoms in early adolescents born very preterm? J Abnorm Child Psychol 45:671–680. 10.1007/s10802-016-0194-0 27573689 10.1007/s10802-016-0194-0

[30] Masten AS, Lucke CM, Nelson KM, Stallworthy IC (2021) Resilience in development and psychopathology: Multisystem perspectives. Annu Rev Clin Psychol 17:521–549. 10.1146/annurev-clinpsy-081219-120307 33534615 10.1146/annurev-clinpsy-081219-120307

[31] Wolke D, Dipl-Psych RM, Meyer R (1999) Cognitive status, language attainment, and prereading skills of 6-year-old very preterm children and their peers: The Bavarian Longitudinal Study. Dev Med Child Neurol 41:94–109. 10.1017/S0012162299000201 10075095 10.1017/s0012162299000201

[32] EryigitMadzwamuse S, Baumann N, Jaekel J et al (2015) Neuro-cognitive performance of very preterm or very low birth weight adults at 26 years. J Child Psychol Psychiatry Allied Discip 56:857–864. 10.1111/jcpp.12358 10.1111/jcpp.12358 25382451

[33] Remschmidt H, Walter R (1990) Psychische Auffälligkeiten bei Schulkindern: Mit deutschen Normen für die Child Behavior Checklist. Verlag für Psychologie, Göttingen

[34] Goodman R (1997) The strengths and difficulties questionnaire: A research note. J Child Psychol Psychiatry 38:581–586. 10.1111/J.1469-7610.1997.TB01545.X 9255702 10.1111/j.1469-7610.1997.tb01545.x

[35] Goodman A, Lamping DL, Ploubidis GB (2010) When to use broader internalising and externalising subscales instead of the hypothesised five subscales on the strengths and difficulties questionnaire (SDQ): Data from british parents, teachers and children. J Abnorm Child Psychol 38:1179–1191. 10.1007/s10802-010-9434-x 20623175 10.1007/s10802-010-9434-x

[36] Achenbach TM (1997) Manual for the young adult self-report and young adult behavior checklist. University of Vermont, Department of Psychiatry

[37] Klasen H, Woerner W, Wolke D et al (2000) Comparing the German versions of the strengths and difficulties questionnaire (SDQ-Deu) and the child behavior checklist. Eur Child Adolesc Psychiatry 9:271–276 11202102 10.1007/s007870070030

[38] Mansolf M, Blackwell CK, Cummings P et al (2022) Linking the child behavior checklist to the strengths and difficulties questionnaire. Psychol Assess 34:233–246. 10.1037/pas0001083 34843282 10.1037/pas0001083 PMC9718585

[39] Fenton TR, Kim JH (2013) A systematic review and meta-analysis to revise the Fenton growth chart for preterm infants. BMC Pediatr 13:59. 10.1186/1471-2431-13-59 23601190 10.1186/1471-2431-13-59 PMC3637477

[40] Bauer A (1988) Ein Verfahren zur Messung des für das Bildungsverhalten relevanten Sozial Status (BRSS)-überarbeitete Fassung. Frankfurt Dtsch Inst für Int Pädagogische Forsch

[41] Kurstjens S, Wolke D (2001) Effects of maternal depression on cognitive development of children over the first 7 years of life. J Child Psychol Psychiatry Allied Discip 42:623–636. 10.1017/S0021963001007296 11464967

[42] Riegel K, Ohrt B, Wolke D, Österlund K (1995) Die Entwicklung gefährdet geborener Kinder bis zum funften Lebensjahr. [The development of children born at risk until their fifth year of life.]

[43] Schmid G, Schreier A, Meyer R, Wolke D (2011) Predictors of crying, feeding and sleeping problems: a prospective study. Child Care Health Dev 37:493–502. 10.1111/j.1365-2214.2010.01201.x 21299592 10.1111/j.1365-2214.2010.01201.x

[44] Esser G, Blanz B, Geisel B, Laucht M (1989) Mannheimer Elterninterview. Mannheim, Ger Beltz

[45] Spanier GB (1976) Measuring dyadic adjustment: New scales for assessing the quality of marriage and similar dyads. J Marriage Fam 38:15–28

[46] Wolke D, Ratschinski G, Ohrt B, Riegel K (1994) The cognitive outcome of very preterm infants may be poorer than often reported: An empirical investigation of how methodological issues make a big difference. Eur J Pediatr 153:906–915. 10.1007/BF01954744 7532133 10.1007/BF01954744

[47] Davis EE (1973) Columbia mental maturity scale. TPGA J 2:147–149

[48] Kiese C, Kozielski PM (1979) Aktiver Wortschatztest für drei- bis sechsjährige Kinder (AWST 3–6) [Active Vocabulary Test for 3–6 Year Olds]. Beltz, Weinheim, Germany

[49] Beery KE, Beery NA (2010) The beery-buktenica developmental test of visual-motor integration. Beery VMI: with supplemental developmental tests of visual perception and motor coordination and stepping stones age norms from birth to age six. Administration, Scoring, and Teaching Man. Pearson

[50] Buss AH, Plomin R (1984) Temperament: Early developing personality traits. Hillsdale, Lawrence Earlbaum Associates

[51] Harter S, Pike R (1984) The pictorial scale of perceived competence and social acceptance for young children. Child Dev 55:1969–1982 6525886

[52] Heuser KM, Jaekel J, Wolke D (2018) Origins and predictors of friendships in 6- to 8-year-old children born at neonatal risk. J Pediatr 193:93-101.e5. 10.1016/j.jpeds.2017.09.072 29241679 10.1016/j.jpeds.2017.09.072

[53] Wolke D (1991) Manual zum Freundschafts- und Familieninterview.[Friendship and Family Interview]. Munich: Bavarian Longitudinal Study, Germany

[54] Debener A, Job AK (2025) Agreement and discrepancies of maternal- and self-reported psychopathology in emerging Adults. J Psychopathol Behav Assess 47:1–14. 10.1007/s10862-024-10177-6

[55] De Reyes AL, Kazdin AE (2005) Informant discrepancies in the assessment of childhood psychopathology: A critical review, theoretical framework, and recommendations for further study. Psychol Bull 131:483–509. 10.1037/0033-2909.131.4.483 16060799 10.1037/0033-2909.131.4.483

[56] Boyle MH, Miskovic V, Van Lieshout R et al (2011) Psychopathology in young adults born at extremely low birth weight. Psychol Med 41:1763–1774. 10.1017/S0033291710002357 21134317 10.1017/S0033291710002357

[57] Hille ETM, Dorrepaal C, Perenboom R et al (2008) Social lifestyle, risk-taking behavior, and psychopathology in young adults born very preterm or with a very low birthweight. J Pediatr 152:793–800 18492518 10.1016/j.jpeds.2007.11.041

[58] Muthén BO, Muthén LK (2000) Integrating person-centered and variable-centered analyses: Growth mixture modeling with latent trajectory classes. Alcohol Clin Exp Res 24:882–891. 10.1111/j.1530-0277.2000.tb02070.x 10888079

[59] van Buuren S, Groothuis-Oudshoorn K (2011) mice: Multivariate imputation by chained equations in R. J Stat Softw 45:1–67. 10.18637/jss.v045.i03

[60] Girard LC (2021) Concomitant trajectories of internalising, externalising, and peer problems across childhood: a person-centered approach. Res Child Adolesc Psychopathol 49:1551–1565. 10.1007/s10802-021-00851-8 34279766 10.1007/s10802-021-00851-8 PMC8557151

[61] Benestad MR, Drageset J, Hufthammer KO et al (2022) Long-term follow-up of self-reported mental health and health-related quality of life in adults born extremely preterm. Early Hum Dev 173. 10.1016/j.earlhumdev.2022.105661 10.1016/j.earlhumdev.2022.105661 36067714

[62] Fuhrmann D, Knoll LJ, Blakemore S-J (2015) Adolescence as a sensitive period of brain development. Trends Cogn Sci 19:558–566. 10.1016/j.tics.2015.07.008 26419496 10.1016/j.tics.2015.07.008

[63] Adjei NK, Jonsson KR, Straatmann VS et al (2024) Impact of poverty and adversity on perceived family support in adolescence: findings from the UK Millennium Cohort Study. Eur Child Adolesc Psychiatry. 10.1007/s00787-024-02389-8 38353677 10.1007/s00787-024-02389-8 PMC11424735

[64] Smith LK, Draper ES, Manktelow BN et al (2007) Socioeconomic inequalities in very preterm birth rates. Arch Dis Child Fetal Neonatal Ed 92:11–14. 10.1136/adc.2005.090308 10.1136/adc.2005.090308 PMC2675287 16595590

[65] Heikkilä K, Metsälä J, Pulakka A et al (2023) Preterm birth and the risk of multimorbidity in adolescence: a multiregister-based cohort study. Lancet Public Heal 8:e680–e690. 10.1016/S2468-2667(23)00145-7 10.1016/S2468-2667(23)00145-7 37633677

[66] Bilgin A, Mendonca M, Wolke D (2018) Preterm birth/low birth weight and markers reflective of wealth in adulthood: a meta-analysis. Pediatrics 142:1–13. 10.1542/peds.2017-3625 10.1542/peds.2017-3625 29875181

[67] Marlow N, Ni Y, Lancaster R et al (2021) No change in neurodevelopment at 11 years after extremely preterm birth. Arch Dis Childhood-Fetal Neonatal Ed 106:418–424 33504573 10.1136/archdischild-2020-320650

[68] Cheong JLY, Spittle AJ, Burnett AC et al (2020) Have outcomes following extremely preterm birth improved over time? In: Seminars in Fetal and Neonatal Medicine. Elsevier, p 101114 10.1016/j.siny.2020.101114 32451304

[69] Rinne GR, Mahrer NE, Guardino CM et al (2023) Childhood family stress modifies the association between perinatal stressful life events and depressive symptoms. J Fam Psychol 37:432–442. 10.1037/fam0001076 36996242 10.1037/fam0001076 PMC10238650

[70] Rothbart MK, Ahadi SA (1994) Temperament and the development of personality. J Abnorm Psychol 103:55–66. 10.1037/0021-843X.103.1.55 8040481 10.1037//0021-843x.103.1.55

[71] Prior M, Smart D, Sanson ANN, Oberklaid F (2000) Does shy-inhibited temperament in childhood lead to anxiety problems in adolescence? J Am Acad Child Adolesc Psychiatry 39:461–468 10761348 10.1097/00004583-200004000-00015

[72] Belsky J, Pluess M (2009) Beyond diathesis stress: Differential susceptibility to environmental influences. Psychol Bull 135:885–908. 10.1037/a0017376 19883141 10.1037/a0017376

[73] Slagt M, Dubas JS, Deković M, van Aken MAG (2016) Differences in sensitivity to parenting depending on child temperament: A meta-analysis. Psychol Bull 142:1068–1110. 10.1037/bul0000061 27513919 10.1037/bul0000061

[74] Van Lieshout RJ, Boyle MH, Saigal S et al (2015) Mental health of extremely low birth weight survivors in their 30s. Pediatrics 135:452–459. 10.1542/peds.2014-3143 25667243 10.1542/peds.2014-3143

[75] Treyvaud K, Inder TE, Lee KJ et al (2012) Can the home environment promote resilience for children born very preterm in the context of social and medical risk? J Exp Child Psychol 112:326–337. 10.1016/j.jecp.2012.02.009 22480454 10.1016/j.jecp.2012.02.009

[76] den Haan PJ, de Kroon MLA, van Dokkum NH et al (2019) Risk factors for emotional and behavioral problems in moderately-late preterms. PLoS One 14:1–11. 10.1371/journal.pone.0216468 10.1371/journal.pone.0216468 PMC6497297 31048855

[77] Tancredy CM, Fraley RC (2006) The nature of adult twin relationships: An attachment-theoretical perspective. J Pers Soc Psychol 90:78–93. 10.1037/0022-3514.90.1.78 16448311 10.1037/0022-3514.90.1.78

[78] Rees P, Gale C, Battersby C et al (2025) Intraventricular hemorrhage and survival, multimorbidity, and neurodevelopment. JAMA Netw Open 8:e2452883–e2452883. 10.1001/jamanetworkopen.2024.52883 39761048 10.1001/jamanetworkopen.2024.52883 PMC11704976

[79] Larsen J, Holland J, Kochhar P et al (2024) Comparing the prevalence of psychiatric disorders in cohorts of children born extremely preterm in 1995 and 2006: the EPICure studies. JAACAP Open 2:217–228. 10.1016/j.jaacop.2024.02.005 39239392 10.1016/j.jaacop.2024.02.005 PMC11372438

[80] Burstein O, Aryeh T, Geva R (2024) Neonatal care and developmental outcomes following preterm birth: A systematic review and meta-analysis. Dev Psychol. 10.1037/dev0001844 39480317 10.1037/dev0001844

[81] Wolke D, Waylen A, Samara M et al (2009) Selective drop-out in longitudinal studies and non-biased prediction of behaviour disorders. Br J Psychiatry 195:249–256 19721116 10.1192/bjp.bp.108.053751 PMC2802508

[82] Hille ETM, Elbertse L, Gravenhorst JB et al (2005) Nonresponse bias in a follow-up study of 19-year-old adolescents born as preterm infants. Pediatrics 116:e662–e666 16263980 10.1542/peds.2005-0682

