# Supplementary material for: Developmental trajectories of internalizing problems among individuals born very preterm/very low birthweight: early risk and resilience factors
Source: Eur Child Adolesc Psychiatry. 2025 May 15;34(10):3197–208. doi: 10.1007/s00787-025-02736-3 (PMC12592270; doi:10.1007/s00787-025-02736-3)

**Supplemental Information**

**Developmental Trajectories of Internalizing Problems among Individuals Born Very Preterm/Very Low Birthweight: Early Risk and Resilience Factors**

Yanlin Zhou, Peter Bartmann, Nicole Tsalacopoulos, Dieter Wolke^*^

**Table S1.** Comparison of internalizing scores between complete and incomplete datasets

|  | **Complete data**  (4 time points, n=197) | **With missings**  (<4 time points, n=171) | ***t*-value** | ***p*-value** |
| --- | --- | --- | --- | --- |
| **6 years** | 7.60 ± 5.57 | 7.80 ± 5.79 | 0.106 | .745 |
| **8 years** | 7.38 ± 5.27 | 7.69 ± 5.52 | 0.261 | .610 |
| **13 years** | 4.47 ± 3.53 | 5.17 ± 3.68 | 2.824 | .094 |
| **26 years** | 6.78 ± 6.29 | 6.84 ± 6.31 | 0.003 | .953 |

**Table S2.** Sociodemographic characteristics of the final sample and dropout participants.

|  | **Final sample** (n=368) | **Dropouts**  (n=140) | **χ² or *F*** | ***p*-value** |
| --- | --- | --- | --- | --- |
| **Biological sex** (Male) | 52.2% | 50.7% | 0.087 | .769 |
| **Gestational age** (weeks, M±SD) | 30.49 ± 2.29 | 30.26 ± 2.38 | 1.046 | .307 |
| **Birthweight** (grams, M±SD) | 1295 ± 307 | 1328 ± 300 | 1.218 | .270 |
| **Hospitalization duration** (days, M±SD) | 77.89 ± 35.10 | 81.79 ± 43.69 | 1.092 | .297 |
| **Maternal age** (years, M±SD) | 28.6 ± 5.02 | 28.48 ± 5.64 | 0.052 | .819 |
| **Parental marital status** (Married and cohabiting) | 82.5% | 74.8% | 3.542 | .060 |
| **Maternal mental health** (no problems) | 77.9% | 81.2 % | 0.735 | .692 |
| **Socioeconomic status** |  |  | 20.11 | **<.001** |
| Low | 36% | 58.6% |  |  |
| Mid | 43.1% | 26.6% |  |  |
| High | 21% | 14.8% |  |  |

| **Table S3.** Clinical categories of internalizing scores by trajectory and timepoint in the VPT/VLBW group (n=368) | | | | |
| --- | --- | --- | --- | --- |
|  |  | **Consistently Low**  (n = 227) | **Increasing**  (n = 80) | **Decreasing**  (n = 61) |
| **6 years internalizing Z score** | | |  |  |
| *Normal (< +1 SD)* | | 212 (93.4%) | 60 (75.0%) | 9 (14.8%) |
| *Borderline (+1SD ~+ 2SD)* | | 7 (3.1%) | 15 (18.8%) | 28 (45.9%) |
| *Clinical (> +2SD)* | | 0 (0%) | 1 (1.3%) | 21 (34.4%) |
| *Missing* | | 8 (3.5%) | 4 (5%) | 3 (4.9%) |
| **8 years internalizing Z score** | | |  |  |
| *Normal (< +1 SD)* | | 199 (87.7%) | 56 (70%) | 23 (37.7%) |
| *Borderline (+1SD ~+ 2SD)* | | 5 (2.2%) | 11 (13.8%) | 20 (32.8%) |
| *Clinical (> +2SD)* | | 0 (0%) | 7 (8.8%) | 13 (21.3%) |
| *Missing* | | 23 (10.1%) | 6 (7.5%) | 5 (8.2%) |
| **13 years internalizing Z score** | | |  |  |
| *Normal (< +1 SD)* | | 177 (78%) | 36 (45%) | 31 (50.8%) |
| *Borderline (+1SD ~+ 2SD)* | | 10 (4.4%) | 27 (33.8%) | 12 (19.7%) |
| *Clinical (> +2SD)* | | 2 (0.9%) | 9 (11.2%) | 8 (13.1%) |
| *Missing* | | 38 (16.7%) | 8 (10%) | 10 (16.4%) |
| **26 years internalizing Z score** | | |  |  |
| *Normal (< +1 SD)* | | 150 (66.1%) | 2 (2.5%) | 31 (50.8%) |
| *Borderline (+1SD ~+ 2SD)* | | 0 (0%) | 40 (50%) | 5 (8.2%) |
| *Clinical (> +2SD)* | | 0 (0%) | 19 (23.8%) | 0 (0%) |
| *Missing* | | 77 (33.9%) | 19 (23.8%) | 25 (41%) |
| *Note.* Standard deviations from the whole sample-based z-scores were used as cutoffs for clinical classification. This approach aligns with the established clinical scoring interpretations of CBCL and SDQ. | | | | |

| **Table S4.** Univariable and multivariable association between early risk and resilience factors and increasing and decreasing trajectories of internalizing problems | | | | | | | | | | | | | | | |
| --- | --- | --- | --- | --- | --- | --- | --- | --- | --- | --- | --- | --- | --- | --- | --- |
|  | **Increasing** | | | | | | |  | **Decreasing** | | | | | | |
|  | **Univariable** | | |  | **Multivariable** | | |  | **Univariable** | | |  | **Multivariable** | | |
|  | OR | 95% CI | *p* |  | OR | 95% CI | *p* |  | OR | 95% CI | *p* |  | OR | 95% CI | *p* |
| ***Neonatal and Biological Factors*** | | | | | | | | | | | | | | | |
| Sex (Female) | 0.88 | [0.53, 1.47] | .635 |  | 0.92 | [0.51, 1.65] | .781 |  | 0.82 | [0.46, 1.44] | .481 |  | 1.29 | [0.6, 2.81] | .515 |
| Gestational age (per week) | **0.84** | **[0.74, 0.94]** | **.003** |  | **0.83** | **[0.70, 0.99]** | **.046** |  | **0.82** | **[0.72, 0.94]** | **.004** |  | 0.99 | [0.77, 1.27] | .929 |
| Birthweight z score (per SD) | 1.19 | [0.96, 1.47] | .111 |  | 0.97 | [0.71, 1.33] | .862 |  | **1.39** | **[1.09, 1.78]** | **.008** |  | **1.61** | **[1.05, 2.46]** | **.029** |
| Multiple birth | **0.44** | **[0.22, 0.87]** | **.018** |  | **0.43** | **[0.20, 0.93]** | **.032** |  | 0.61 | [0.30, 1.22] | .163 |  | **0.31** | **[0.11, 0.87]** | **.027** |
| IVH stage 3/4 | 1.23 | [0.46, 3.33] | .678 |  | 0.87 | [0.28, 2.67] | .806 |  | 1.36 | [0.47, 3.93] | .574 |  | 0.72 | [0.15, 3.51] | .682 |
| BPD | 1.34 | [0.80, 2.24] | .267 |  | 0.92 | [0.49, 1.71] | .782 |  | 1.45 | [0.81, 2.58] | .206 |  | 1.31 | [0.55, 3.12] | .538 |
| Ever breastfed | 0.72 | [0.38, 1.37] | .319 |  | 0.63 | [0.30, 1.30] | .213 |  | 0.92 | [0.46, 1.85] | .821 |  | 1.11 | [0.44, 2.81] | .829 |
| ***Family and Parenting Factors*** | | | | | | | | | | | | | | | |
| High SES (vs. Low) | 0.54 | [0.29, 0.99] | .052 |  | **0.41** | **[0.18, 0.98]** | **.045** |  | 0.59 | [0.26, 1.31] | .194 |  | 0.92 | [0.30, 2.88] | .891 |
| Middle SES (vs. Low) | 1.85 | [0.90, 3.83] | .093 |  | 1.10 | [0.54, 2.25] | .795 |  | 0.59 | [0.32, 1.13] | .111 |  | 1.40 | [0.48, 4.10] | .536 |
| Maternal mental health | 0.99 | [0.51, 1.91] | .971 |  | 1.29 | [0.62, 2.67] | .498 |  | 0.99 | [0.73, 1.34] | .941 |  | 0.88 | [0.37, 2.07] | .768 |
| Parent–infant relationship problems@5m | 1.20 | [0.94, 1.54] | .138 |  | 1.05 | [0.79, 1.40] | .752 |  | **0.51** | **[0.27, 0.97]** | **.039** |  | **0.60** | **[0.38, 0.94]** | **.030** |
| Family adversity@5m/20m/56m | 1.05 | [0.95, 1.17] | .339 |  | 1.05 | [0.92, 1.21] | .45 |  | **1.30** | **[1.16, 1.45]** | **<.001** |  | **1.32** | **[1.11, 1.57]** | **<.001** |
| Psychosocial stress@5m/20m/56m | 1.05 | [0.96, 1.15] | .313 |  | 1.04 | [0.93, 1.17] | .483 |  | **1.19** | **[1.10, 1.30]** | **<.001** |  | 1.14 | [1.00, 1.30] | .050 |
| Lower parenting quality@6y | 1.15 | [0.84, 1.59] | .379 |  | 1.27 | [0.90, 1.81] | .178 |  | **1.83** | **[1.28, 2.63]** | **.001** |  | 1.38 | [0.87, 2.19] | .171 |
| Partnership quality@6y | 0.98 | [0.97, 1.00] | .058 |  | 0.99 | [0.98, 1.01] | .460 |  | **0.97** | **[0.96, 0.99]** | **<.001** |  | 0.98 | [0.96, 1.00] | .075 |
| ***Child Neurodevelopment and Temperament*** | | | | | | | | | | | | | | | |
| [IQ@56m](mailto:IQ@56m) | 0.82 | [0.64, 1.06] | .123 |  | 1.21 | [0.80, 1.83] | .364 |  | **0.68** | **[0.52, 0.88]** | **.004** |  | 0.94 | [0.52, 1.70] | .844 |
| Neurosensory impairment@56m/6y | **2.02** | **[1.17, 3.49]** | **.012** |  | **2.47** | **[1.03, 5.92]** | **.044** |  | 1.77 | [0.96, 3.26] | .068 |  | 0.86 | [0.26, 2.90] | .813 |
| Regulatory problems@6y | 0.88 | [0.56, 1.37] | .569 |  | 0.89 | [0.56, 1.42] | .619 |  | 1.28 | [0.85, 1.93] | .235 |  | 1.16 | [0.65, 2.07] | .606 |
| EAS temperament-Activity@6y | 1.00 | [0.93, 1.07] | .931 |  | 1.02 | [0.94, 1.10] | .625 |  | 0.93 | [0.87, 1.00] | .062 |  | 0.96 | [0.87, 1.06] | .443 |
| EAS temperament-Effortful control@6y | 0.95 | [0.89, 1.01] | .076 |  | 0.96 | [0.89, 1.03] | .245 |  | **0.90** | **[0.84, 0.97]** | **.004** |  | **0.87** | **[0.79, 0.96]** | **.005** |
| EAS temperament-Shyness@6y | 1.01 | [0.99, 1.04] | .373 |  | 1.02 | [0.99, 1.05] | .203 |  | **1.08** | **[1.05, 1.11]** | **<.001** |  | **1.07** | **[1.03, 1.12]** | **.002** |
| EAS temperament-Socialbility@6y | 0.98 | [0.92, 1.04] | .439 |  | 1.02 | [0.93, 1.11] | .695 |  | **0.90** | **[0.84, 0.96]** | **.002** |  | 0.97 | [0.87, 1.09] | .629 |
| EAS temperament-Emotionality@6y | 1.06 | [1.00, 1.13] | .056 |  | 1.02 | [0.95, 1.10] | .524 |  | **1.22** | **[1.13, 1.32]** | **<.001** |  | **1.20** | **[1.09, 1.32]** | **<.001** |
| ***Social Contexts Factors*** | | | | | | | | | | | | | | | |
| Peer relationships@6y | 0.60 | [0.35, 1.02] | .057 |  | 1.31 | [0.65, 2.63] | .457 |  | **0.49** | **[0.27, 0.89]** | **.019** |  | 2.08 | [0.83, 5.25] | .121 |
| Friendship quality@6y | 0.94 | [0.85, 1.04] | .257 |  | 0.96 | [0.85, 1.08] | .479 |  | **0.85** | **[0.76, 0.96]** | **.007** |  | 0.90 | [0.77, 1.06] | .205 |
| Bully victimization@6y | 1.05 | [0.89, 1.23] | .569 |  | 0.97 | [0.81, 1.16] | .762 |  | **1.17** | **[0.99, 1.37]** | **.049** |  | 1.01 | [0.82, 1.25] | .918 |
| Neighborhood child friendliness@6y | 0.81 | [0.56, 1.19] | .283 |  | 0.73 | [0.49, 1.08] | .117 |  | 0.89 | [0.58, 1.36] | .596 |  | 0.68 | [0.40, 1.17] | .164 |
| ***Note.*** Consistently low trajectory was the reference group. IVH, intraventricular hemorrhage. BPD, bronchopulmonary dysplasia. | | | | | | | | | | | | | | | |

| **Table S5.** Multivariable association between early factors and increasing and decreasing trajectories of internalizing problems | | | | | | | | |
| --- | --- | --- | --- | --- | --- | --- | --- | --- |
|  |  | **Increasing** | | |  | **Decreasing** | | |
|  |  | OR | 95% CI | *p* |  | OR | 95% CI | *p* |
| ***Neonatal and Biological Factors*** |  |  |  |  |  |  |  |  |
| Sex (Female) |  | 0.94 | [0.47, 1.86] | .936 |  | 1.46 | [3.91, 0.54] | .453 |
| Gestational age (per week) |  | **0.83** | **[0.72, 0.94]** | **.045** |  | 0.98 | [0.72, 1.35] | .92 |
| Birthweight z score (per SD) |  | 0.96 | [0.67, 1.37] | .800 |  | **1.67** | **[1.01, 2.77]** | **.049** |
| Multiple birth |  | **0.37** | **[0.16, 0.87]** | **.023** |  | **0.24** | **[0.07, 0.83]** | **.024** |
| IVH stage 3/4 |  | 0.96 | [0.27, 3.47] | .962 |  | 0.98 | [7.52, 0.13] | .983 |
| BPD |  | 0.89 | [0.45, 1.77] | .890 |  | 1.22 | [0.44, 3.40] | .698 |
| Ever breastfed |  | 0.59 | [0.27, 1.31] | .591 |  | 1.14 | [0.36, 3.56] | .824 |
| ***Family and Parenting Factors*** |  |  |  |  |  |  |  |  |
| High SES (vs. Low) |  | **0.36** | **[0.16, 0.80]** | **.013** |  | 0.86 | [0.23, 3.23] | .822 |
| Middle SES (vs. Low) |  | 0.40 | [0.15, 1.09] | .073 |  | 0.68 | [0.24, 1.96] | .485 |
| Maternal mental health |  | 0.81 | [0.34, 1.97] | .646 |  | 0.62 | [0.22, 1.72] | .357 |
| Parent–infant relationship problems@5m |  | 0.93 | [0.64, 1.35] | .692 |  | **0.58** | **[0.33, 0.99]** | **.041** |
| Family adversity@5m/20m/56m |  | 1.07 | [0.92, 1.26] | .389 |  | **1.42** | **[1.15, 1.76]** | **.001** |
| Psychosocial stress@5m/20m/56m |  | 0.98 | [0.86, 1.12] | .772 |  | 1.10 | [0.94, 1.27] | .23 |
| Lower parenting quality@6y |  | 1.20 | [0.80, 1.81] | .385 |  | 1.24 | [0.69, 2.25] | .472 |
| Partnership quality@6y |  | 0.99 | [0.97, 1.01] | .119 |  | 0.97 | [0.94, 1.00] | .053 |
| ***Child Neurodevelopment and Temperament*** |  |  |  |  |  |  |  |  |
| [IQ@56m](mailto:IQ@56m) |  | 1.14 | [0.67, 1.92] | .624 |  | 0.78 | [0.39, 1.56] | .478 |
| Neurosensory impairment@56m/6y |  | **2.23** | **[1.23, 4.03]** | **.008** |  | 0.80 | [0.20, 3.15] | .749 |
| Regulatory problems@6y |  | 0.75 | [0.43, 1.30] | .303 |  | 1.01 | [0.50, 2.06] | .976 |
| EAS temperament-Activity@6y |  | 0.97 | [0.88, 1.07] | .540 |  | 0.93 | [0.83, 1.05] | .233 |
| EAS temperament-Effortful control@6y |  | 0.98 | [0.90, 1.06] | .604 |  | 0.91 | [0.79, 1.05] | .193 |
| EAS temperament-Shyness@6y |  | 1.03 | [0.98, 1.08] | .280 |  | **1.11** | **[1.05, 1.18]** | **<.001** |
| EAS temperament-Socialbility@6y |  | 1.04 | [0.94, 1.15] | .419 |  | 1.04 | [0.90, 1.20] | .624 |
| EAS temperament-Emotionality@6y |  | 1.02 | [0.94, 1.12] | .610 |  | **1.21** | **[1.07, 1.36]** | **.002** |
| ***Social Contexts Factors*** |  |  |  |  |  |  |  |  |
| Peer relationships@6y |  | 0.97 | [0.42, 2.24] | .935 |  | 2.19 | [0.72, 6.65] | .165 |
| Friendship quality@6y |  | 0.95 | [0.81, 1.12] | .548 |  | 0.90 | [0.75, 1.08] | .259 |
| Bully victimization@6y |  | 1.06 | [0.86, 1.31] | .580 |  | 1.09 | [0.84, 1.42] | .501 |
| Neighborhood child friendliness@6y |  | 0.99 | [0.61, 1.62] | .971 |  | 0.91 | [0.48, 1.72] | .764 |
| ***Note.*** This model accounts for family cluster-robust standard errors and weights for individual probabilities of being assigned to the most likely classes. Consistently low trajectory was the reference group. IVH, intraventricular hemorrhage. BPD, bronchopulmonary dysplasia. | | | | | | | | |

**Figure S1. Confirmatory Factor Analysis of Parent-Reported Internalizing Problems**

***Figure S1A: 6 yrs CBCL***

χ^2^ = 1457.140, *df* = 431, *p* <.001; CFI = 0.762, RMSEA = 0.047, SRMR = 0.050.

**
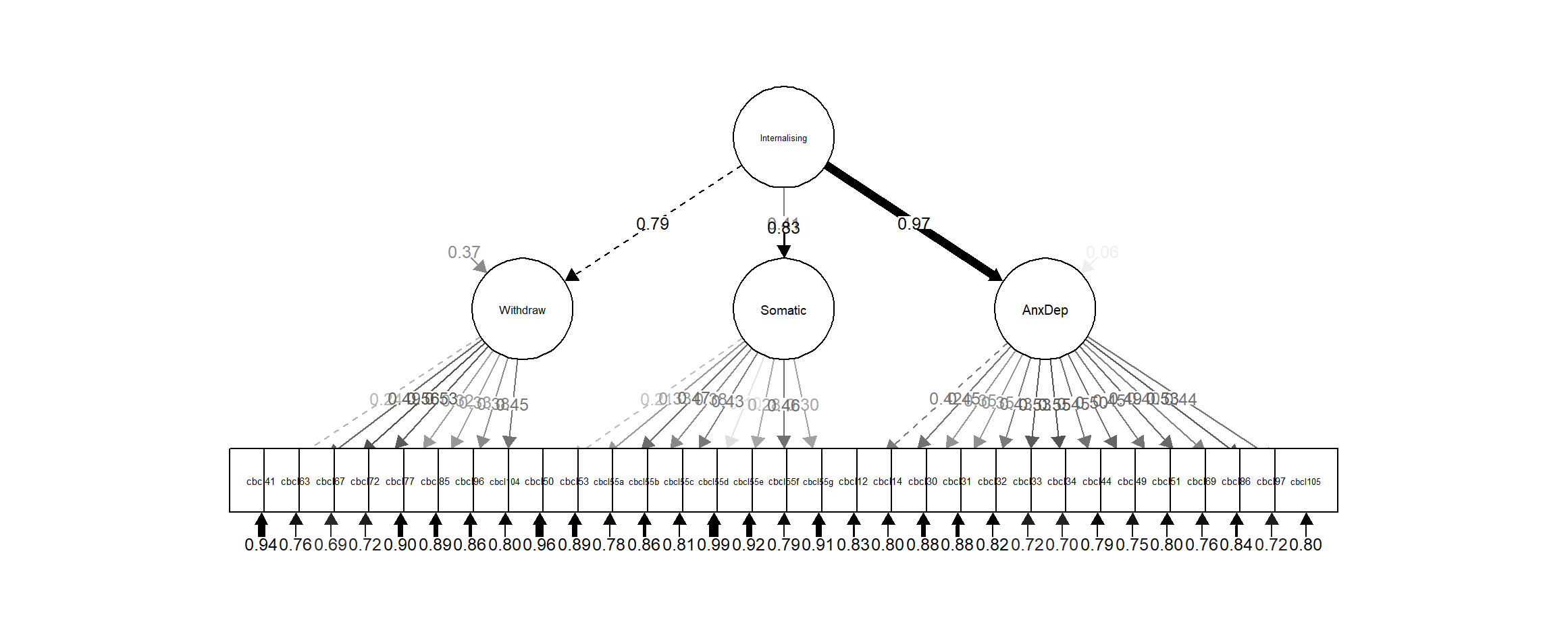
**

Latent Variables:

Estimate Std.Err z-value P(>|z|) Std.lv Std.all

Withdraw =~

cbcl41 1.000 0.130 0.238

cbcl63 1.549 0.256 6.041 0.000 0.202 0.493

cbcl67 2.085 0.312 6.692 0.000 0.271 0.560

cbcl72 2.411 0.384 6.283 0.000 0.314 0.526

cbcl77 0.708 0.139 5.097 0.000 0.092 0.322

cbcl85 1.452 0.282 5.143 0.000 0.189 0.329

cbcl96 1.059 0.195 5.424 0.000 0.138 0.377

cbcl104 1.180 0.196 6.013 0.000 0.154 0.451

Somatic =~

cbcl50 1.000 0.047 0.209

cbcl53 2.737 0.895 3.059 0.002 0.128 0.327

cbcl55a 2.585 0.903 2.864 0.004 0.121 0.471

cbcl55b 2.406 0.790 3.046 0.002 0.112 0.379

cbcl55c 1.954 0.593 3.295 0.001 0.091 0.432

cbcl55d 0.593 0.308 1.926 0.054 0.028 0.098

cbcl55e 2.849 0.936 3.042 0.002 0.133 0.280

cbcl55f 3.251 1.040 3.126 0.002 0.152 0.459

cbcl55g 1.442 0.459 3.140 0.002 0.067 0.301

AnxDep =~

cbcl12 1.000 0.199 0.417

cbcl14 1.403 0.134 10.494 0.000 0.280 0.449

cbcl30 0.437 0.063 6.887 0.000 0.087 0.349

cbcl31 1.070 0.127 8.431 0.000 0.213 0.353

cbcl32 1.014 0.103 9.872 0.000 0.202 0.429

cbcl33 1.090 0.099 11.024 0.000 0.217 0.529

cbcl34 1.039 0.105 9.880 0.000 0.207 0.549

cbcl44 1.147 0.104 11.021 0.000 0.229 0.454

cbcl49 1.363 0.142 9.578 0.000 0.272 0.495

cbcl51 0.554 0.072 7.725 0.000 0.110 0.447

cbcl69 1.383 0.152 9.115 0.000 0.276 0.487

cbcl86 0.973 0.108 8.969 0.000 0.194 0.398

cbcl97 1.046 0.089 11.726 0.000 0.208 0.527

cbcl105 1.025 0.097 10.610 0.000 0.204 0.444

Internalizing =~

Withdraw 1.000 0.792 0.792

Somatic 0.187 0.066 2.823 0.005 0.412 0.412

AnxDep 1.881 0.420 4.481 0.000 0.972 0.972

***Figure S1B: 8 yrs CBCL***

χ^2^ = 1369.325, df = 431, p <.001; CFI = 0.758, RMSEA = 0.039, SRMR = 0.053.


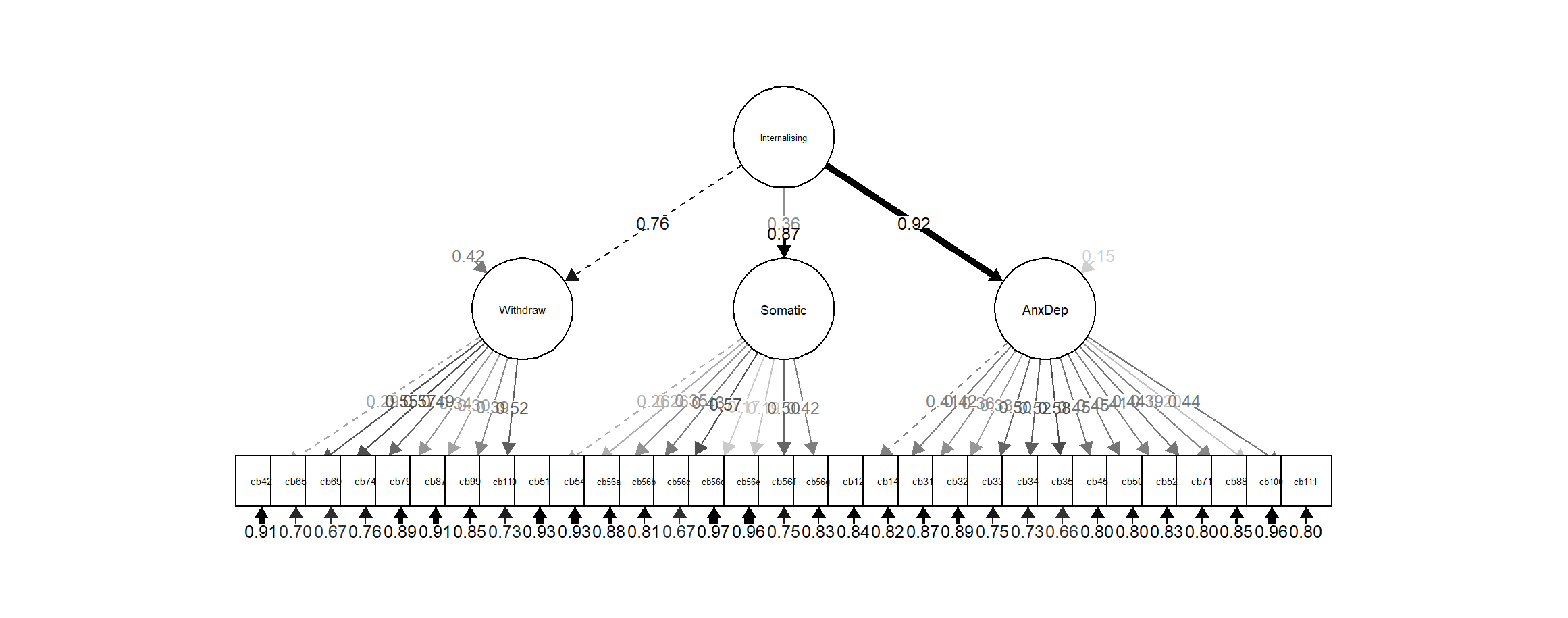


Latent Variables:

Estimate Std.Err z-value P(>|z|) Std.lv Std.all

Withdraw =~

cb42 1.000 0.154 0.294

cb65 1.197 0.163 7.341 0.000 0.184 0.550

cb69 1.782 0.245 7.273 0.000 0.275 0.573

cb74 1.812 0.218 8.303 0.000 0.279 0.490

cb79 0.593 0.117 5.062 0.000 0.091 0.337

cb87 1.113 0.204 5.464 0.000 0.172 0.296

cb99 1.056 0.150 7.054 0.000 0.163 0.391

cb110 0.965 0.129 7.459 0.000 0.149 0.520

Somatic =~

cb51 1.000 0.069 0.259

cb54 1.428 0.365 3.908 0.000 0.098 0.255

cb56a 1.277 0.489 2.611 0.009 0.088 0.351

cb56b 2.534 0.643 3.939 0.000 0.174 0.430

cb56c 2.254 0.592 3.807 0.000 0.155 0.570

cb56d 0.843 0.360 2.338 0.019 0.058 0.169

cb56e 1.269 0.528 2.406 0.016 0.087 0.190

cb56f 2.641 0.769 3.434 0.001 0.182 0.500

cb56g 1.222 0.486 2.514 0.012 0.084 0.415

AnxDep =~

cb12 1.000 0.185 0.405

cb14 1.355 0.140 9.681 0.000 0.250 0.419

cb31 0.473 0.072 6.567 0.000 0.087 0.363

cb32 1.121 0.154 7.290 0.000 0.207 0.332

cb33 1.375 0.124 11.121 0.000 0.254 0.497

cb34 1.133 0.114 9.971 0.000 0.209 0.521

cb35 1.428 0.137 10.393 0.000 0.264 0.582

cb45 1.284 0.134 9.563 0.000 0.237 0.447

cb50 1.281 0.150 8.514 0.000 0.237 0.451

cb52 0.588 0.078 7.543 0.000 0.109 0.408

cb71 1.276 0.150 8.515 0.000 0.236 0.445

cb88 0.961 0.130 7.368 0.000 0.177 0.388

cb100 0.063 0.031 2.069 0.039 0.012 0.196

cb111 1.136 0.123 9.260 0.000 0.210 0.442

Internalizing =~

Withdraw 1.000 0.758 0.758

Somatic 0.214 0.073 2.931 0.003 0.363 0.363

AnxDep 1.453 0.305 4.766 0.000 0.920 0.920

***Figure S1C: 13 yrs SDQ***

χ^2^ = 77.828, df = 33, p <.001; CFI = 0.957, RMSEA = 0.046, SRMR = 0.038.


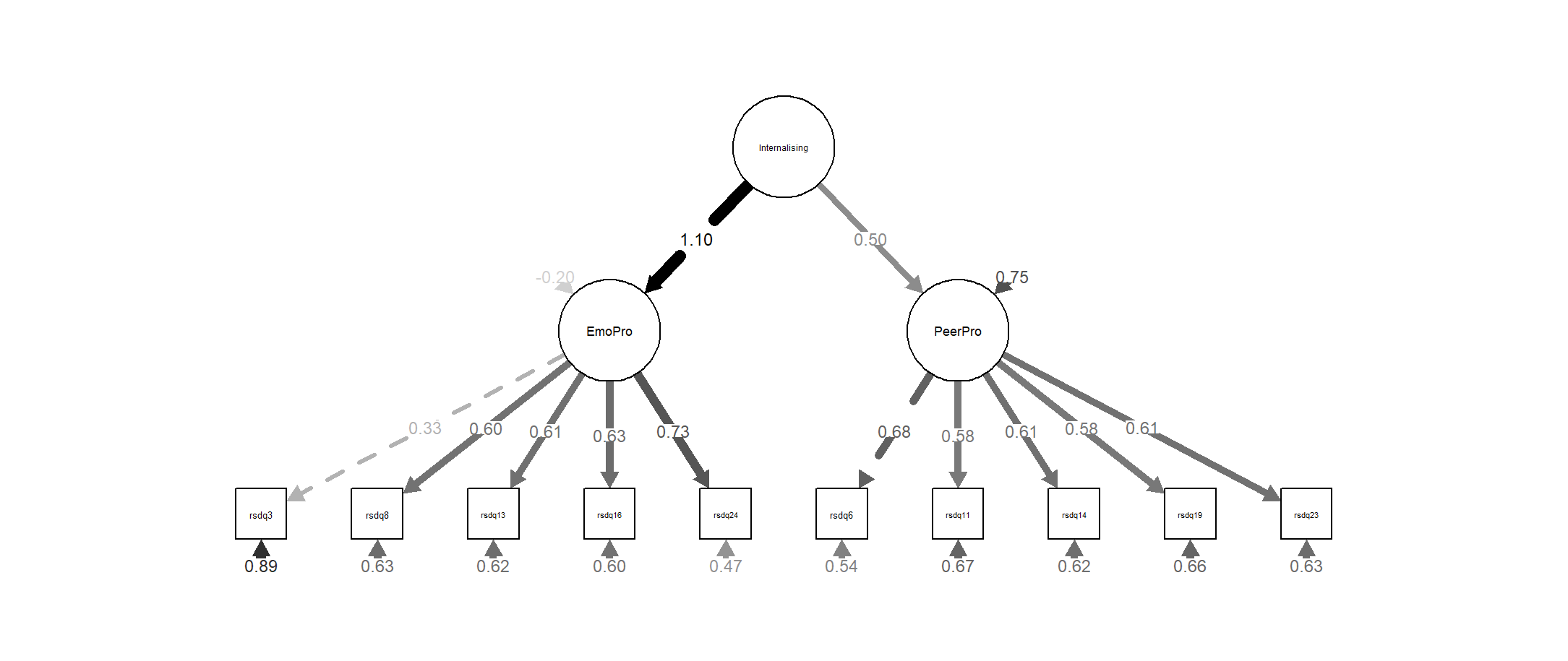


Latent Variables:

Estimate Std.Err z-value P(>|z|) Std.lv Std.all

EmoPro =~

rsdq3 1.000 0.198 0.331

rsdq8 1.962 0.343 5.724 0.000 0.388 0.605

rsdq13 1.617 0.254 6.370 0.000 0.320 0.615

rsdq16 2.217 0.457 4.851 0.000 0.438 0.632

rsdq24 2.251 0.463 4.862 0.000 0.445 0.731

PeerPro =~

rsdq6 1.000 0.439 0.680

rsdq11 0.588 0.065 9.030 0.000 0.258 0.576

rsdq14 0.611 0.064 9.581 0.000 0.268 0.614

rsdq19 0.701 0.077 9.155 0.000 0.308 0.580

rsdq23 0.847 0.077 10.949 0.000 0.372 0.611

Internalizing =~

EmoPro 1.000 1.096 1.096

PeerPro 1.006 0.084 11.951 0.000 0.496 0.496

***Figure S1D: 26 yrs YABCL***

χ^2^ = 551.723, df = 150, p <.001; CFI = 0.839, RMSEA = 0.074, SRMR = 0.073.


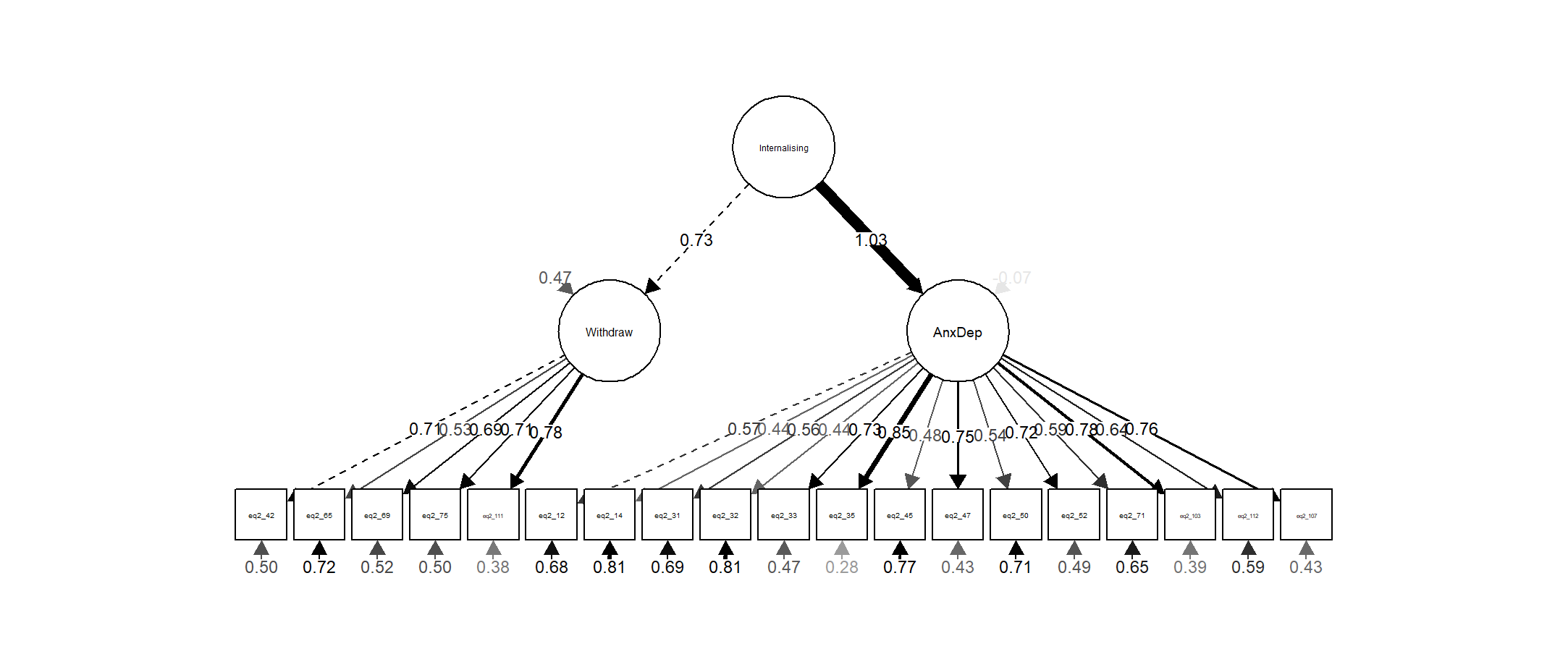


Latent Variables:

Estimate Std.Err z-value P(>|z|) Std.lv Std.all

Withdraw =~

eq2_42 1.000 0.389 0.706

eq2_65 0.452 0.137 3.306 0.001 0.176 0.532

eq2_69 1.078 0.166 6.481 0.000 0.419 0.691

eq2_75 1.079 0.151 7.122 0.000 0.419 0.707

eq2_111 1.011 0.060 16.740 0.000 0.393 0.785

AnxDep =~

eq2_12 1.000 0.271 0.566

eq2_14 0.457 0.126 3.644 0.000 0.124 0.439

eq2_31 0.685 0.236 2.905 0.004 0.185 0.558

eq2_32 1.206 0.219 5.512 0.000 0.326 0.439

eq2_33 1.258 0.205 6.140 0.000 0.341 0.727

eq2_35 1.663 0.270 6.155 0.000 0.450 0.847

eq2_45 1.068 0.176 6.057 0.000 0.289 0.479

eq2_47 1.671 0.345 4.847 0.000 0.452 0.753

eq2_50 0.952 0.238 3.993 0.000 0.258 0.539

eq2_52 1.123 0.224 5.006 0.000 0.304 0.717

eq2_71 1.142 0.281 4.059 0.000 0.309 0.594

eq2_103 1.622 0.261 6.214 0.000 0.439 0.780

eq2_112 1.614 0.276 5.836 0.000 0.437 0.641

eq2_107 1.665 0.291 5.716 0.000 0.451 0.758

Internalizing =~

Withdraw 1.000 0.730 0.730

AnxDep 0.987 0.564 1.748 0.080 1.034 1.034

**Figure S2: Bivariate Correlation Analysis of Early Factor Variables**


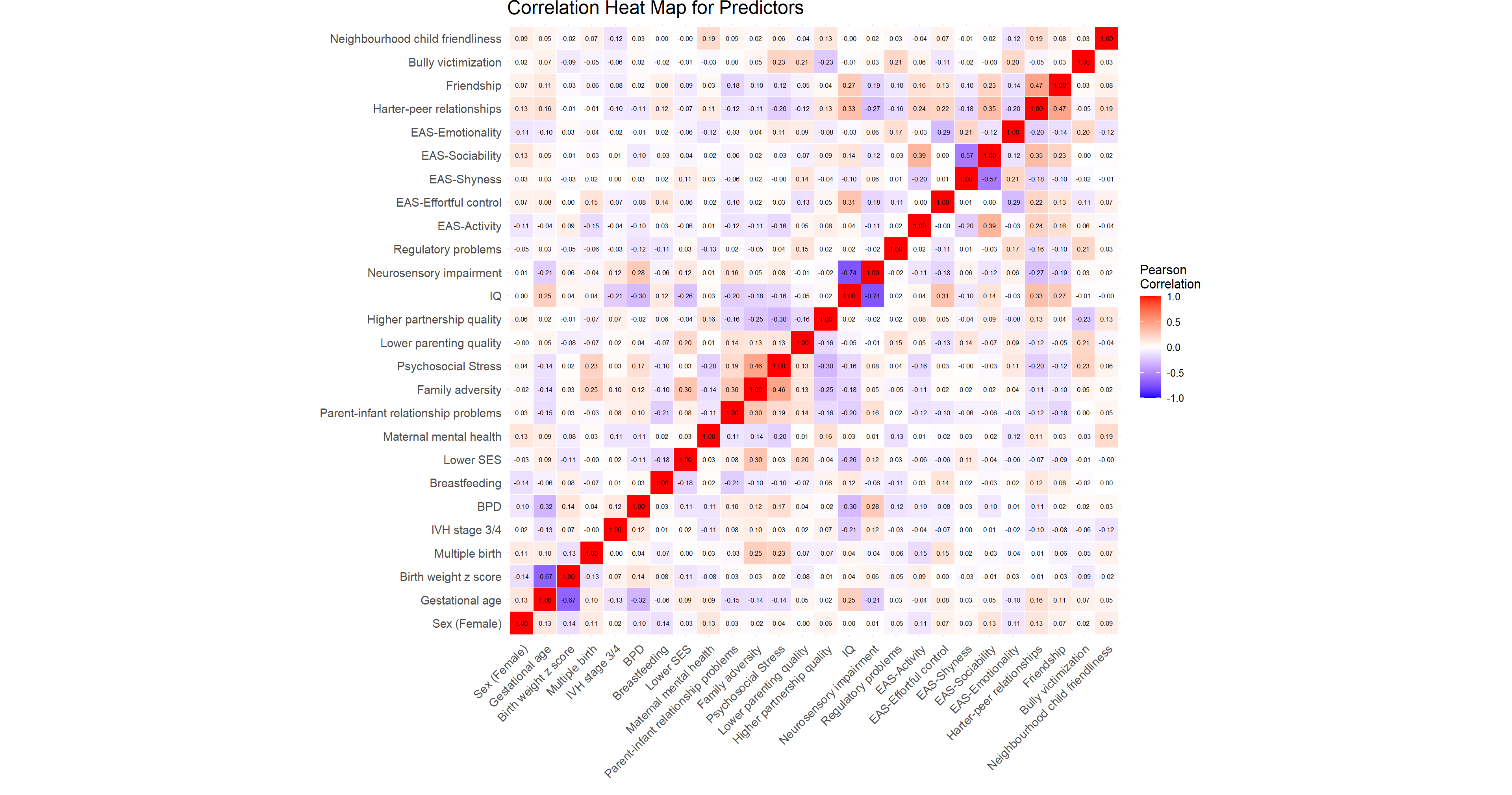


**Figure S3: Missing Data Patterns for Early Factors**


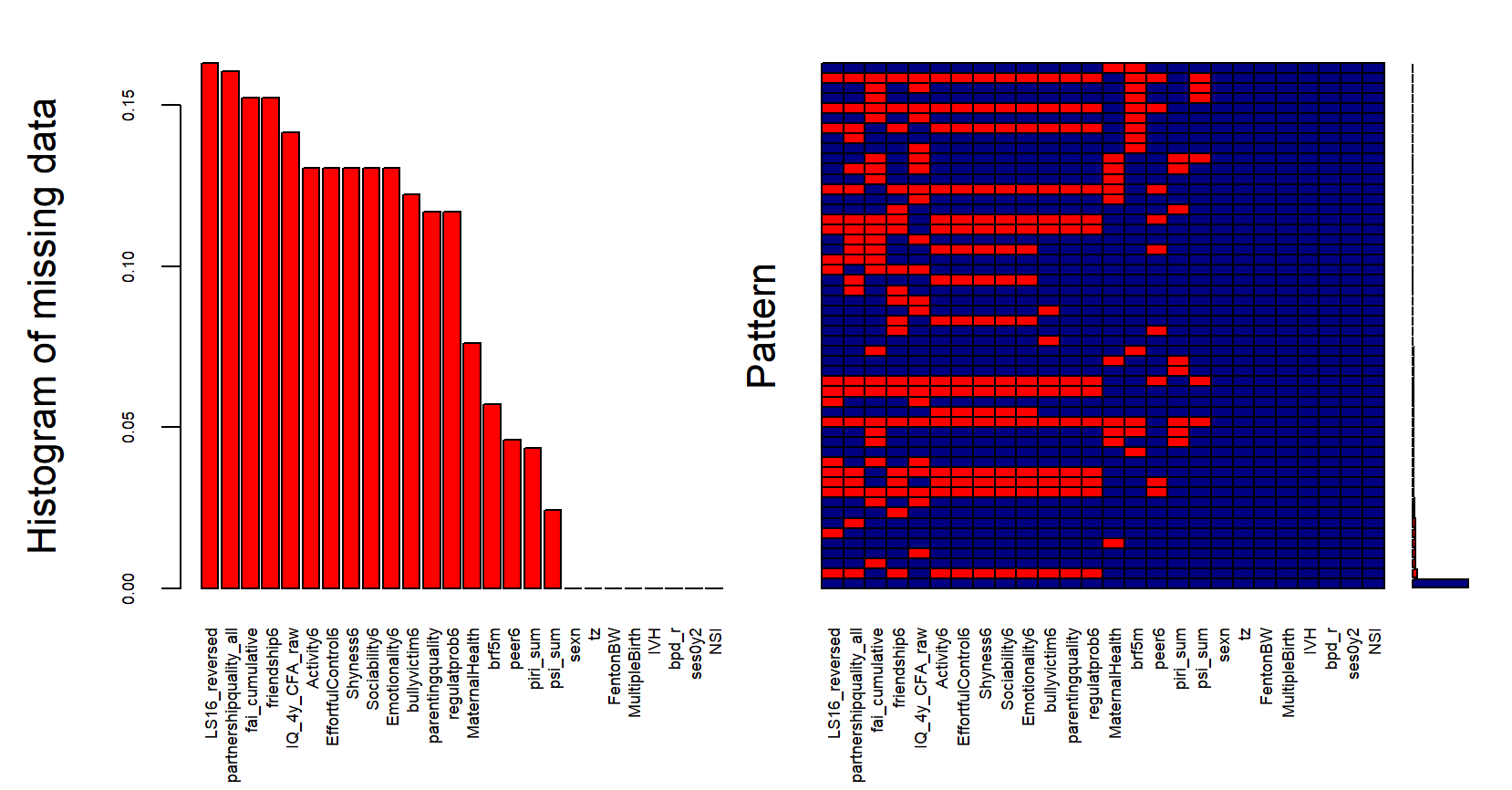

Supplement: Supplementary file 1 — Supplementary file1 (DOCX 1043 KB) [file 787_2025_2736_MOESM1_ESM.docx]
